# Comprehensive genetic analysis of 961 unrelated Duchenne Muscular Dystrophy patients: Focus on diagnosis, prevention and therapeutic possibilities

**DOI:** 10.1371/journal.pone.0232654

**Published:** 2020-06-19

**Authors:** Shalini H. Kumar, Kalpana Athimoolam, Manikandan Suraj, Mary Shoba Das Christu Das, Aparna Muralidharan, Divya Jeyam, Jaicy Ashokan, Priya Karthikeyan, Ragav Krishna, Arati Khanna-Gupta, Lakshmi Bremadesam Raman

**Affiliations:** Molecular Diagnostics, Counseling, Care and Research Centre (MDCRC), Royal CareSuper Speciality Hospital, Neelambur, Coimbatore, Tamil Nadu, India; University of Bonn, Institute of Experimental Hematology and Transfusion Medicine, GERMANY

## Abstract

Recently DNA sequencing analysis has played a vital role in the unambiguous diagnosis of clinically suspected patients with Duchenne Muscular Dystrophy (DMD). DMD is a monogenic, X-linked, recessive, degenerative pediatric neuromuscular disorder affecting males, invariably leading to fatal cardiopulmonary failure. Early and precise diagnosis of the disease is an essential part of an effective disease management strategy as care guidelines and prevention through counseling need to be initiated at the earliest particularly since therapies are now available for a subset of patients. In this manuscript we report the *DMD* gene mutational profiles of 961 clinically suspected male DMD patients, 99% of whom were unrelated. We utilized a molecular diagnostic approach which is cost-effective for most patients and follows a systematic process that sequentially involves identification of hotspot deletions using mPCR, large deletions and duplications using MLPA and small insertions/ deletions and point mutations using an NGS muscular dystrophy gene panel. Pathogenic *DMD* gene mutations were identified in 84% of patients. Our data compared well with the frequencies and distribution of deletions and duplications reported in the *DMD* gene in other published studies. We also describe a number of rare in-frame mutations, which appeared to be enriched in the 5’ proximal hotspot region of the *DMD* gene. Furthermore, we identified a family with a rare non-contiguous deletion mutation in the *DMD* gene where three males were affected and two females were deemed carriers. A subset of patients with mutations in the *DMD* gene who are likely to benefit therapeutically from new FDA and EMA approved drugs were found in our cohort. Given that the burden of care for DMD patients invariably falls on the mothers, particularly in rural India, effective genetic counseling followed by carrier screening is crucial for prevention of this disorder. We analyzed the carrier status of consented female relatives of 463 probands to gauge the percentage of patients with familial disease. Our analysis revealed 43.7% of mothers with *DMD* gene mutations. Our comprehensive efforts, involving complete genetic testing coupled with compassionate genetic counseling provided to DMD patients and their families, are intended to improve the quality of life of DMD patients and to empower carrier females to make informed reproductive choices to impede the propagation of this deadly disease.

## Introduction

Duchenne muscular dystrophy (DMD; OMIM: 310200) is a common X-Linked recessive degenerative neuromuscular disorder [1], which afflicts males with an incidence of 1 in 3300–5000 live births [2,3]. Patients with DMD begin to show symptoms between the ages of 2–5 years, these include calf muscle hypertrophy, frequent falls, walking on toes, waddling gait, difficulty in climbing stairs, Gower’s sign and progressive muscular degeneration. Patients become non-ambulatory and wheelchair dependent by the age of twelve and most often succumb to cardiopulmonary failure in their early 20s, although treatment with glucocorticoids extends mobility and lifespan [4]. Mutations in the *DMD* gene encoding the dystrophin protein are responsible for DMD as well as a milder form of the disease referred to as Becker Muscular dystrophy (BMD; OMIM: 300376). *DMD* is the largest gene in humans, being 2.4 Mb in size harboring 79 exons. The full length transcript gives rise to a 427kD protein (3685 amino acids) that is composed of four domains: an amino-terminal actin binding domain (ABD), a central rod domain with spectrin-like repeats, a cysteine-rich domain, and a unique carboxy-terminal domain. *DMD* mRNA is expressed mainly in the muscles, heart and brain [5,6]

Within the muscle fiber, dystrophin protein associates with the dystrophin associated protein complex (DAPC), a multi-protein complex harboring α- and β-dystrobrevins, dystroglycans, sarcoglycans, sarcospan, syntrophins, and laminins. DAPC links the intracellular actin cytoskeleton to the extracellular matrix, providing structural stability during muscular activity. Loss of the dystrophin protein as a result of mutations in the *DMD* gene leads to loss of membrane integrity. Chronic bouts of myofiber degeneration and regeneration then lead to aberrant activation of the NF-κB (nuclear factor kappa B) inflammatory pathway resulting in progressive membrane damage, ultimately leading to muscular degeneration [7].

Thus far no definitive curative treatment options are available for patients with DMD (Rev in [8]). The use of glucocorticoid anti-inflammatory drugs such as prednisone or deflazacort to alleviate inflammation, represent the current standard of care. While corticosteroids have been associated with partial improvement in muscle strength, cardiopulmonary function and ambulation, serious side effects, including increased bone fragility, stunting of growth, weight gain, adrenal suppression and delayed puberty are the biggest drawbacks [9]. More recently, a first in class dissociative steroidal anti-inflammatory drug, vamorolone, showed an improved safety profile compared to prednisone. Additionally, vamorolone demonstrated anti-inflammatory activity in a phase IIa trial, presumably through stabilizing the myofiber membrane leading to improved muscle strength [10]. This promising drug however awaits further testing in patients and final FDA approval.

The most encouraging therapeutic options available to date thus far involve an exon-skipping approach and a stop codon read through approach. The former enables DMD patients to produce dystrophins such as found in Becker patients, albeit at significantly lower levels, leading to a slowdown in disease progression, while the latter attempts to reestablish the functional integrity of the protein [11,12]. Both these approaches require very precise identification of the mutational status of the *DMD* gene in DMD/BMD patients.

In the absence of curative treatment options, the early diagnosis of patients with suspected DMD is vital to implementing effective disease management strategies, keeping the quality of life of the patient in mind. In this regard, genetic counseling for patients and their families must be emphasized. In providing a humane and compassionate platform through which disease ontology, disease progression and its inevitable outcome is disseminated to patients and their families, the importance of genetic counseling should not be underestimated. Genetic counseling also stresses the importance of carrier testing in female relatives of DMD patients [13], and serves to help families understand and manage the impact of having one or more DMD patients in the family.

In this report we present the first comprehensive genetic analysis of a large cohort of 961clinically suspected, 99% unrelated DMD patients at our center (MDCRC) in Coimbatore in the southern Indian state of Tamil Nadu. We have effectively implemented a region-appropriate, molecular diagnostic algorithm, which sequentially utilizes multiplex PCR, multiplex ligation dependent probe amplification (MLPA) [14,15] and next generation sequencing (NGS) to detect genetic variants in the 79 exons of the *DMD* gene. As expected, the majority of the patients had deletion (66%) and duplication (7.5%) mutations in the *DMD* gene. A striking pattern was observed in DMD patients defying the “reading frame rule,” where patients with in-frame *DMD* gene mutations demonstrated an enrichment of deletion and duplication mutations in the proximal hotspot region of the *DMD* gene. Additionally, we found patients with 114 novel *DMD* gene deletion, duplication and point mutations, and describe a family with three affected males and two carrier females harboring a rare non-contiguous mutation. NGS analysis further identified patients with small mutations and provided unequivocal clarity in distinguishing DMD versus non-DMD muscular dystrophies. Our mutational analyses identified a subset of patients who would likely benefit from recently approved FDA and EMA drugs. Genetic analyses also demonstrated that carrier testing, especially in mothers with an older DMD child, coupled with genetic counseling could provide informed family planning options to prevent the propagation of this deadly disease.

## Methods

### Inclusion and exclusion of patients

Nine hundred and sixty one clinically suspected DMD/BMD male patients, 99% of who were unrelated, from the state of Tamil Nadu (TN), India were received at MDCRC, (a non- profit organization dedicated to diagnosis, care and counseling of patients with Muscular Dystrophies: www.mdcrcindia.org) between 2006 to 2013, for molecular diagnosis. Patients were referred to MDCRC through an MDCRC Community Genetics initiative which relied on a community outreach program to identify, diagnose and counsel patients in rural districts of TN state. Some patients were also referred by hospitals and clinics in the state. This initiative has been very successful and will be detailed extensively elsewhere (manuscript in preparation).

Initial clinical diagnosis of these patients at the community primary health care level relied mainly on symptoms, with special emphasis on age at onset of disease, frequency of falls, waddling gait, calf muscle hypertrophy, difficulty climbing stairs, Gower’s sign and age at loss of ambulation. Patients with one or more of these clinical symptoms were identified as clinically suspected DMD patients and were included in our study (S1 Table). Two exclusion criteria were considered in our study, these included: male patients above the age of 35 years and females with DMD like symptoms. An informed written consent was obtained from each patient or parent in the local language (Tamil), where appropriate, prior to inclusion in the study. The Institutional Review Board of Molecular Diagnostics, Counseling, Care & Research Centre (MDCRC) reviewed and approved this study. The approval number is MDCRC/03/IEC-DMD016.

### Genetic analysis of patient samples for diagnoses of DMD

**Fig 1:**
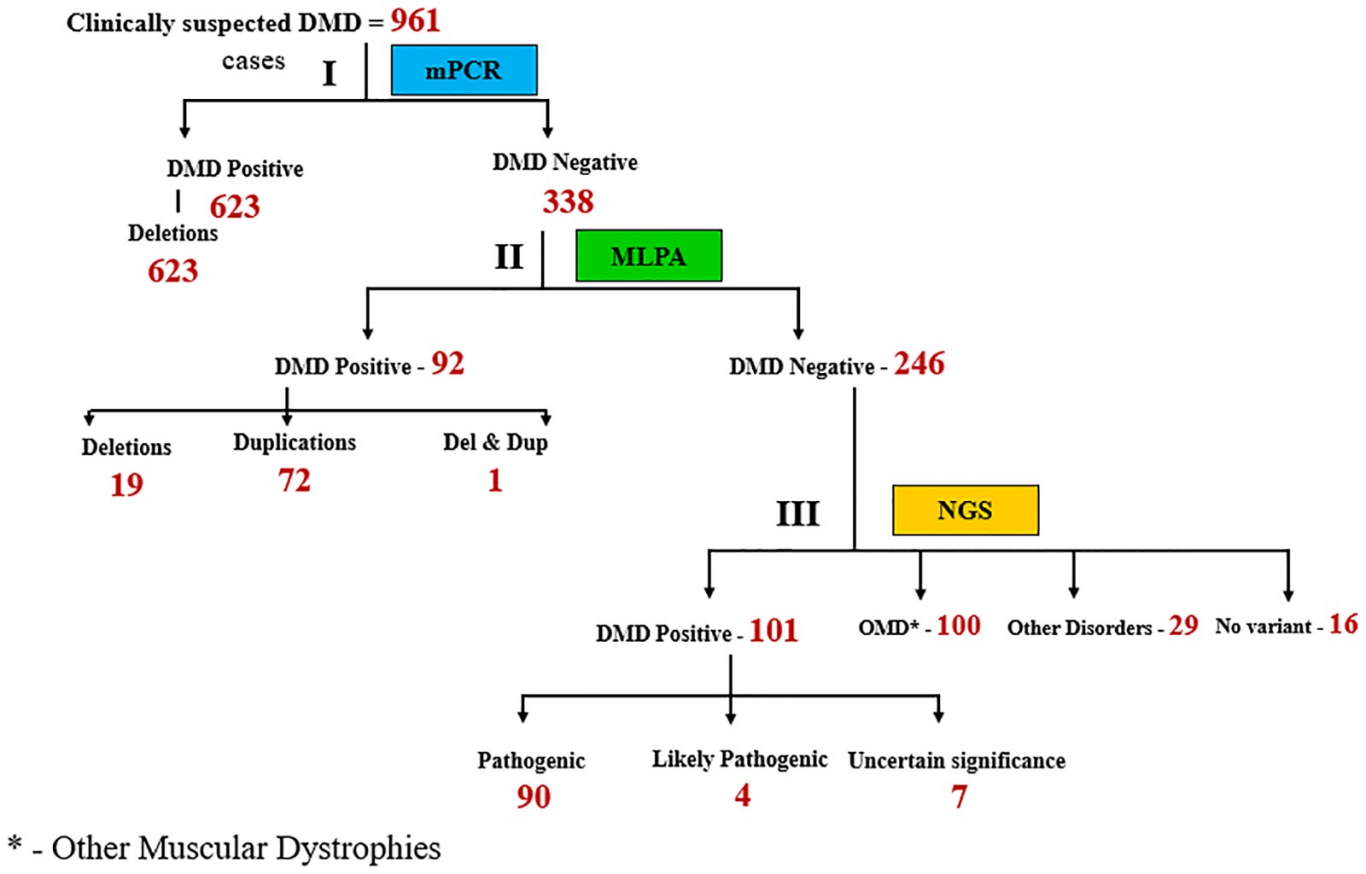
Diagnostic strategy used to identify mutations in 961 suspected DMD patients. DNA isolated from patient samples was subjected to the following steps: Step I involved multiplex PCR (mPCR), step II involved MLPA and step III involved NGS (Next Gen sequencing). The numbers in red indicate number of patients. OMD: Other muscular dystrophies.

Three milliliters of blood samples were collected from clinically suspected DMD patients in EDTA vacutainers. Genomic DNA was next extracted using a simple desalting protocol as previously described [16]. DNA was stored at -20°C until further use. The molecular diagnostic workup of the genomic DNA was analyzed as outlined by the algorithm in Fig 1.

### Multiplex PCR

mPCR was designed to detect any deletion in the hot spot region of the *DMD* gene covering 30 exons as we have previously described [17]. Briefly, multiplex PCR analysis was performed for 30 exons at the central and 5’end hot spot region of the *DMD* gene. The primers for the 30 exons were obtained from the *www*.*dmd*.*nl* website. Multiplex PCR was done in 6 sets each consisting of 4–6 exons. The exons tested were: 1, 3, 4, 6, 8, 12, 13, 16, 17, 19, 20, 21, 22, 32, 34, 41, 42, 43, 44, 45, 46, 47, 48, 49, 50, 51, 52, 53, 55 and 60. PCR was carried out as previously described [15]. In the event that exons 53 or 55 were found to be deleted, testing of exon 54was performed. Reaction products were separated on a 2% agarose gel and analyzed for the presence of deletions. Patient samples negative for mutations in the DMD gene by mPCR, or where the borders of the identified mutations were unclear (BNC: borders not clear), were subjected to MLPA analysis.

### Multiplex ligation-dependent probe amplification (MLPA)

MLPA analysis was carried out using PO34 and PO35 probes purchased commercially from MRC, Holland (Amsterdam, The Netherlands). All procedures were carried out according to the manufacturer’s recommendations as previously described [15]. Amplification products were run on an ABI PRISM 3100 Genetic Analyzer (Applied Biosystems, USA) and data obtained analysed using Genemapper 3.7.

### Next generation sequence analysis

NGS analysis was performed at MedGenome Labs (Bangalore, India) on DNA extracted by the method mentioned above on samples that were negative by mPCR and MLPA. To identify the causal gene variant, targeted next generation sequencing (NGS) was performed. Targeted sequencing libraries were prepared using the Roche Nimblegen SeqCap kit (Pleasnton, CA, USA). Biotinylated oligonucleotide capture probes, also called baits, were designed for the targeted exons in our muscular dystrophy and congenital myopathy gene panel for NGS analysis (S2 Table), and were provided with the kit and used to enrich the region of interest (targeted gene regions) by hybridization. The workflow involved shearing of DNA, repairing ends, adenylation of 3’ ends, followed by adapter ligation. At each step the products were purified. The adapter sequences were added onto the ends of DNA fragments to generate libraries. The resulting adaptor-ligated libraries were purified, quantified and hybridized to the target- specific biotinylated capture library. Following hybridization, the targeted molecules were captured on streptavidin beads. The resulting enriched DNA libraries were next amplified using Illumina adapter specific primers, followed by purification. The libraries were sequenced on Illumina HiSeq series 4500 to generate 2X150bp sequence reads at 80–100X sequencing on-target depth.

Clinically relevant mutations were annotated using published variants in the literature in comparison to disease databases–ClinVar, OMIM, GWAS, HGMD and SwissVar. Common variants were filtered based on allele frequency in 1000Genome Phase 3, ExAC, EVS, dbSNP147, 1000 Japanese Genome and the MedGenome internal Indian population database (unpublished). Non-synonymous variant calling was calculated using multiple algorithms such as PolyPhen-2, SIFT, Mutation Taster2, Mutation Assessor and LRT in accordance with the guidelines of the American College of Medical Genetics and Genomics (ACMG) [18]. Only non-synonymous and splice site variants found in the muscular dystrophy and congenital myopathy panel genes were used for clinical interpretation. Silent variations that do not result in any change in amino acid in the coding region are not reported. Sanger sequencing using standard protocols on an ABI 3730xl instrument was used to validate the presence of point mutations identified by NGS.

### DMD gene mutation carrier testing in mothers and female relatives

Samples of 463 suspected carrier female relatives (316/463 being mothers) of the above DMD positive probands were taken for carrier testing by MLPA or by Sanger sequencing analysis as deemed appropriate.

## Ethical approval

All procedures performed in this study involving human participants were in accordance with the ethical standards of our institution (MDCRC, Coimbatore, TN, India) and in line with the 1964 Helsinki declaration and its later amendments. The Institutional Review Board of Molecular Diagnostics, Counseling, Care & Research Centre (MDCRC) reviewed and approved this study. The approval number is MDCRC/03/IEC-DMD016. Written consent in the local language (Tamil) was obtained from all patients and/or their parents/guardians.

## Results

### Identification of mutations in the *DMD* gene in clinically suspected DMD patients

A hierarchical molecular diagnostic algorithm was followed for 961 patient samples referred to MDCRC, (Fig 1).

**Fig 2:**
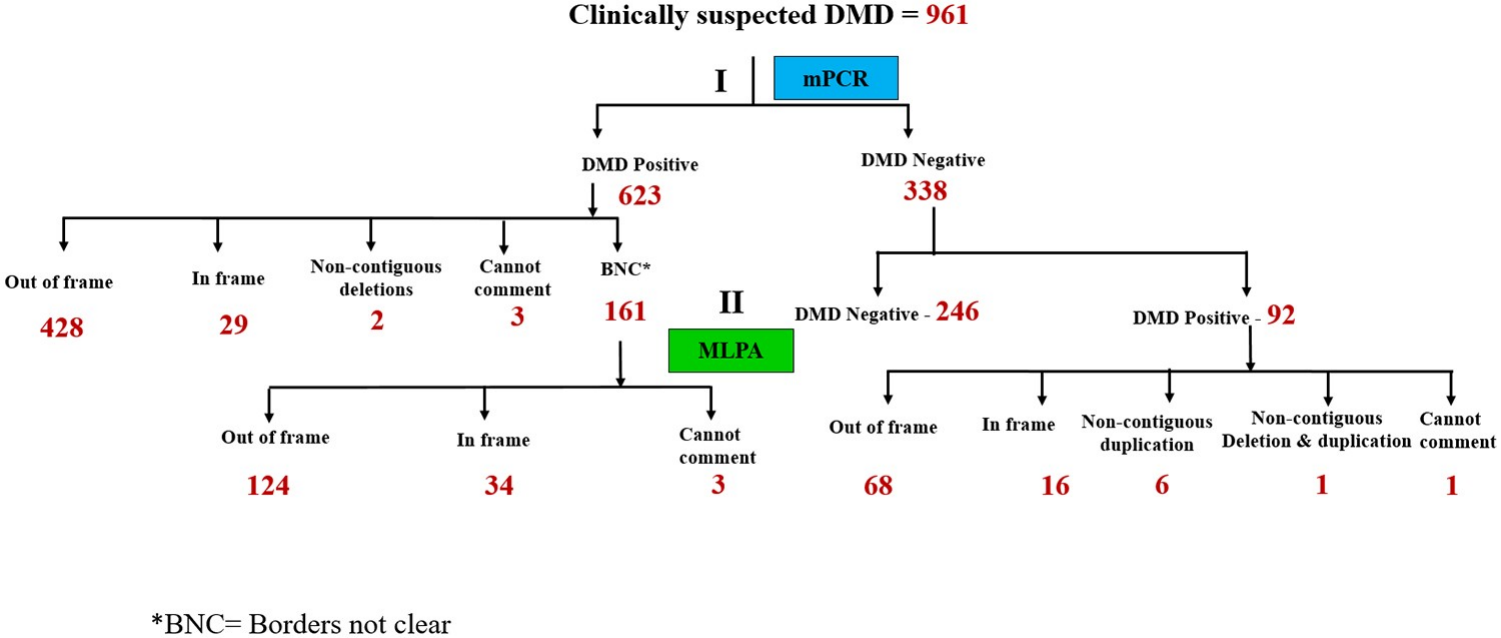
Stratification of DMD positive patients based on mutation type.

All 961 clinically suspected patient DNA samples were initially subjected to multiplex PCR to identify commonly occurring *DMD* “hot spot” deletion mutations covering 30 exons. Based on this analysis, deletions in the *DMD* gene were detected in 623 patients (Fig 1), leaving 338 patients without detected mutations after this round of analysis. DNA from this set of 338 patients was next subjected to MLPA, a well-established method used to detect copy number variation (deletions and/or duplications) in all 79 exons of the *DMD* gene [17]. MLPA analysis revealed an additional 92 patients with *DMD* genemutations (Fig 1). MPCR and MLPA analysis collectively accounted for 74.5% of the 961 patients with deletion and duplication mutations in the *DMD* gene. Detailed analysis of the 623 mPCR positive DMD cases revealed 421 patients with out- of- frame mutations, 28 patients with in-frame mutations, 2 non-contiguous deletion mutations and 3 mutations that remained ambiguous with regard to reading frame due to the involvement of exon 1 deletions, which have previously been shown to contribute to such ambiguity (*www*.*dmd*.*nl*) (Fig 2). Most patients with exon 1 deletions are diagnosed with the milder BMD [19]. Interestingly, two of the three patients with exon 1 deletions in our cohort were over 20 years of age despite having lost ambulation by age 11 (data not shown).

Mutation type of 623 DMD positive patients revealed 161 with boarders not clear (BNC). MLPA analysis of this subset of patients revealed 124 with out-of -frame, 34 with in-frame and 3 patients wherein the *DMD* gene mutation remained unclear. Of the 92 DMD positive patients as assessed by MLPA, 68 harbored out-of-frame, 16 in-frame, 6 non-contiguous duplication, 1 non contiguous deletion and duplication and 1 patient where exon 79 was involved and hence the reading frame could not be predicted.

Following mPCR 161 patients were found to have deletions where the precise start and end of the mutation remained unclear, since mPCR does not screen for all exons in the *DMD* gene (BNC: borders not clear, Table 1 and Fig 2). To fine tune the start and end of each of the 161 *DMD* gene mutations identified by mPCR where the boarders were not clear (BNC, Fig 2), we performed MLPA analysis which classified 124 patients with out-of -frame, 34 with in-frame and 3 patients wherein the *DMD* gene mutation remained unclear (Fig 2 and Table 1). The importance of the deletion borders becomes apparent when predicting the reading frame of the deletion mutation, the knowledge of which is necessary for downstream analysis.

**Table 1:** Detailed sequential analysis of 961 suspected DMD patients leading to confirmed mutational status.

MPCR status	MLPA status	NGS Status	Mutation status	Number of patients
Negative	Negative	NGS Done	No variant	16
Negative	Negative	NGS Done	DMD	98
Negative	Negative	NGS Done	DMD/BMD	2
Negative	Negative	NGS Done	BMD	1
Negative	Negative	NGS Done	OMD	100
Negative	Negative	NGS Done	Other disorders	29
Negative	Positive	DMD	Single Exon Deletion	11
Negative	Positive	DMD	Single Exon Duplication	24
Negative	Positive	DMD	Contiguous Deletion	4
Negative	Positive	DMD	Contiguous Duplication	29
Negative	Positive	BMD	Single Exon Deletion	3
Negative	Positive	BMD	Single Exon Duplication	1
Negative	Positive	BMD	Contiguous Duplication	11
Negative	Positive	Cannot Comment		1
Negative	Positive	Non-Contiguous Duplication		7
Negative	Positive	Non-Contiguous Deletion & Duplication		1
Positive	BNC	MLPA Done	DMD-Single Exon Deletion	7
Positive	BNC	MLPA Done	DMD-Contiguous Deletion	117
Positive	BNC	MLPA Done	BMD-Single Exon Deletion	1
Positive	BNC	MLPA Done	BMD-Contiguous Deletion	33
Positive	BNC	MLPA Done	Cannot Comment	3
Positive	DMD	Single Exon		117
Positive	DMD	Contiguous Deletion		304
Positive	BMD	Single Exon		2
Positive	BMD	Contiguous Deletion		26
Positive	Non-Contiguous Deletion	MLPA Done	Non-Contiguous Deletion	2
Positive	Non-Contiguous Deletion	MLPA Done	Contiguous Deletion	8
Positive	Cannot Comment	MLPA Done	Contiguous Deletion	1
Positive	Cannot Comment	MLPA Done	promoter regions	1
Positive	Cannot Comment			1
**Total**				**961**

Of the 92 DMD positive patients assessed by MLPA (Fig 2), 68 harbored out-of-frame, 16 in-frame mutations, 6 non-contiguous duplications, one with a non contiguous deletion and duplication. One patient harbored an exon 79 mutation and hence the reading frame could not be predicted with certainty (*www*.*dmd*.*nl*).

Table 1 summarizes the mutational status of clinically suspected DMD patients included in our study. This was achieved by sequential analysis of samples by mPCR, MLPA and NGS as outlined in Figs 1 and 2. 98% of 961 patients in our study received an accurate mutational assessment, with the remaining 2% of patients requiring additional DNA sequencing strategies beyond the scope of this study, for mutational conformation. This high level of accuracy in mutation assignment achieved, speaks well for the algorithm we have utilized.

### Deletion mutations in the *DMD* gene reveal the expected mutational hotspots and mutations in patients defying the “reading frame rule”

**Fig 3:**
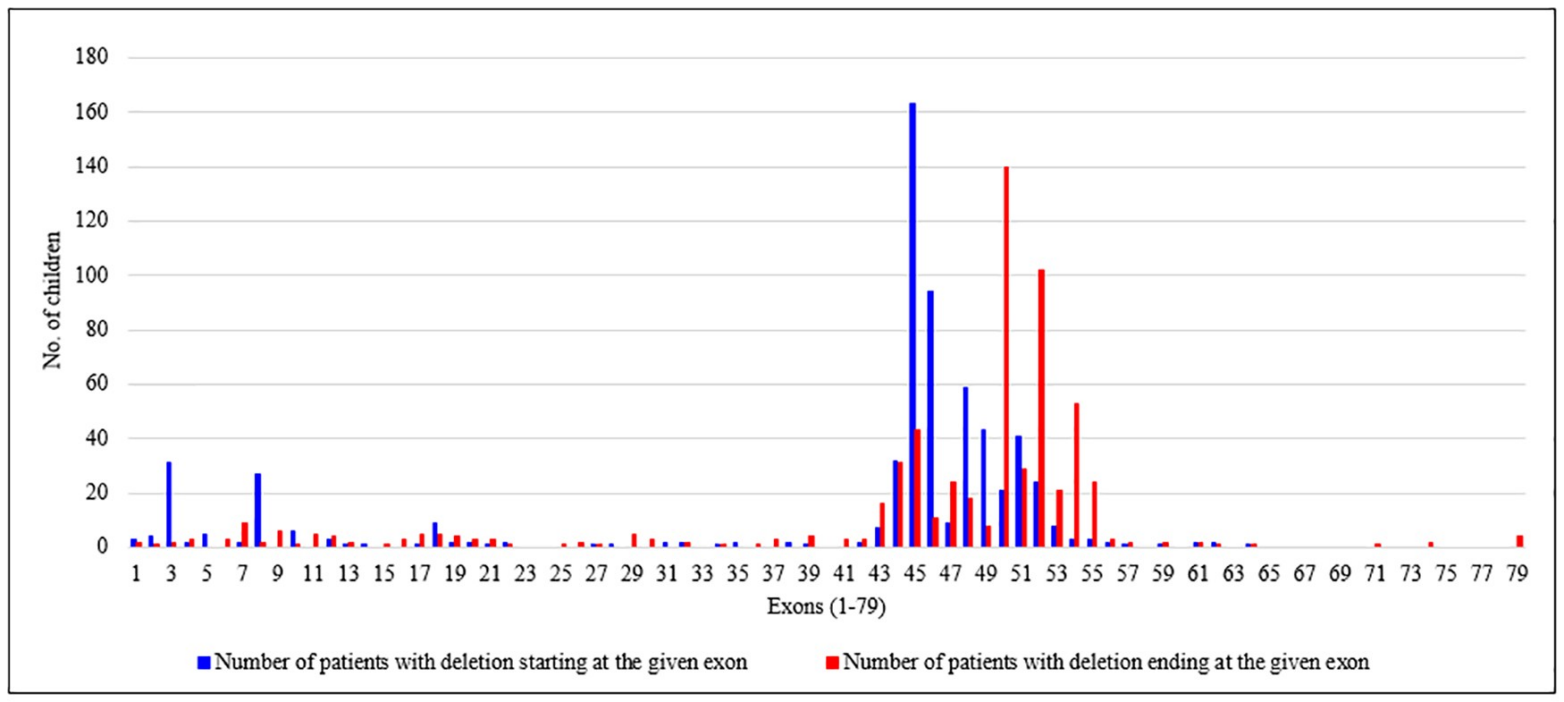
Deletion mutations in the *DMD* gene in 642 patients mapped to two hotspot regions: Proximal (exons 1–22) and distal (exons 43–55). The frequency of deletion mutations in the *DMD* gene has been shown. Blue bars represent patients with deletions beginning in the indicated exon. Red bars represent patients with deletion mutations ending in the indicated exon.

As expected, the majority of deletion mutations found in our cohort of DMD patients were large deletions in the *DMD* gene (Fig 3, and S1 Fig).

Large deletions are known to be the most frequent type of mutation in dystophinopathies, comprising 60–65% of all *DMD* gene mutations found [20,21]. Our mutational analysis using mPCR and MLPA collectively revealed a total of 642 out of 961 patients, accounting for two thirds of the cohort, with deletion mutations in the *DMD* gene (Fig 3). Given that an exon skipping approach has played a crucial role in the development of new therapeutic strategies for DMD in the recent past, we represented all the deletion mutations identified based on their exons of origin (blue bars) and the exons in which the deletions terminated (red bars) (Fig 3). Based on this representation, two previously described hotspots for these deletion mutations were immediately revealed, one ranging from exons 1–22 (proximal hotspot) and the other from exons 43–55 (distal hotspot) (Rev in [22]). Based on our observations, 94% of all deletion mutations began in one of the two hotspot regions while 89.7% ended in these specific regions of the *DMD* gene. In accordance with previously reported findings, our data also showed that nearly 32% of all the deletions described involved exon 45 [20,22]. Mutations involving exon 50 (starting and ending) amounted to 24.9% and exon 52 to 19.5%. Thus, of all the deletion mutations analyzed in our cohort, 76.3% involved exons 45–52.

127 single exon deletions were identified by mPCR in our cohort. These were reconfirmed as authentic single exon mutations by MLPA. However, confirmation that single exon deletions in 14 additional patients identified by MLPA in our cohort, were not small mutations coinciding with one of the MLPA probes, has yet to be validated by NGS.

### Genotype: Phenotype analysis of patients with deletions in the *DMD* gene

**Fig 4:**
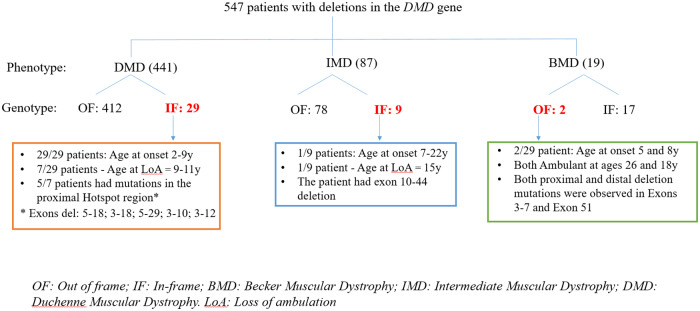
An overview of the genotype: Phenotype correlate of patients with deletions mutations in the *DMD* gene. Patient numbers are indicated. The number of patients defying the reading frame rule are marked in red. OF: out of frame; IF In-frame; BMD: Becker Muscular Dystrophy; IMD: Intermediate Muscular Dystrophy; DMD: Duchenne Muscular Dystrophy; LoA: Loss of ambulation.

We categorized our cohort of 642 patients with deletion mutations based on their clinical presentations, with particular emphasis on the age of onset of symptoms and age of loss of ambulation (Fig 4).

Since patients with deletion mutations in exons 1and 79 cannot be easily assessed for altered reading frame (www.dmd.nl), such patients (7 in number) were removed from analysis of our cohort of 642 patients. Additionally, patients with non-contiguous mutations (2 patients) and ones where phenotype data were unavailable (86 patients), were also eliminated from our analysis. This resulted in 547 patients with documented deletions in the *DMD* gene having the requisite supporting clinical data. The obvious paucity of BMD patients in our cohort (Fig 4; 19 patients) was likely built into our sampling methodology which relied on the notion that patients ambulant at or beyond the age of 16 years were classified as having BMD.

These 547 patients were classified into three categories based on their clinical diagnoses with specific age cutoffs as described below. These included a) DMD: 441 patients, age at onset of symptoms (OS): 0 to 7years; age at loss of ambulation (LoA): ≤12years; b) Intermediate muscular dystrophy (IMD): 87 patients, defined age cutoff for this group: OS:8 to 15years; LoA: 12 to 18years; and c) BMD 19 patients; defined age cutoff for this group: OS:>15years; LoA: >18years (Fig 4).

It has been previously proposed that the clinical severity of DMD is rooted in whether the reading frame of the *DMD* gene is maintained or not. This so called “reading frame rule” postulates that most mutations in the *DMD* gene which disrupt the mRNA translational reading frame (out-of-frame mutations) lead to loss of the dystrophin protein, resulting in DMD. In-frame mutations, on the other hand, lead to the milder BMD, presumably because a truncated but partially functional dystrophin protein is formed. It has been observed, that this rule holds true approximately 90% of the time [16]. While the majority of BMD patients in our cohort had in-frame (IF) mutations (17/19; 89.5%) and DMD patients had out of frame (OF) mutations (412/441; 93.4%), a small percentage of BMD (2/19; 10.5%) and DMD (29 /441; 6.6%) patients defied the reading frame rule (Fig 4, marked in red).

The two unrelated patients had deletions in exons 3–7 and exon 51 in the *DMD* generespectively, remained ambulant at ages 26 and 18, demonstrating BMD characteristics despite the presence of out-of-frame mutations (Fig 4). It is noteworthy that frame-shift mutations in BMD and DMD patients can result in generation of some dystrophin fibres, presumably due to a translation initiation site in exon 8 [23] Of the 87 clinically classified IMD patients, 78 had out of frame mutations and 9 had in-frame mutations. 8/9 patients remained ambulant, with age of onset of symptoms ranging from 8 to 22 years in this cohort.

Of the 29 in-frame DMD patients, 22 developed clinical symptoms between 2–8 years of age. While all 22 patients were ambulant at the time of diagnosis, 7/22 had lost ambulation at the time of writing of this manuscript. All seven patients were under the age of 12 demonstrating typical DMD rather than BMD disease manifestations. The remaining 15 patients were unreachable and their ambulation status could not be assessed.

An additional 7/29 in-frame DMD patients had onset of symptoms typical of most DMD patients, at ages 6–9 years. These patients were non ambulant, having lost ambulation at 9-11years of age. Interestingly, 5/7 of these patients had mutations in the proximal hot-spot region of the *DMD* gene. In summary, all patients in this cohort behaved much like classic DMD patients despite having in-frame mutations in the *DMD* gene.

### Genotype: Phenotype analysis of patients with duplications in the *DMD* gene

**Fig 5:**
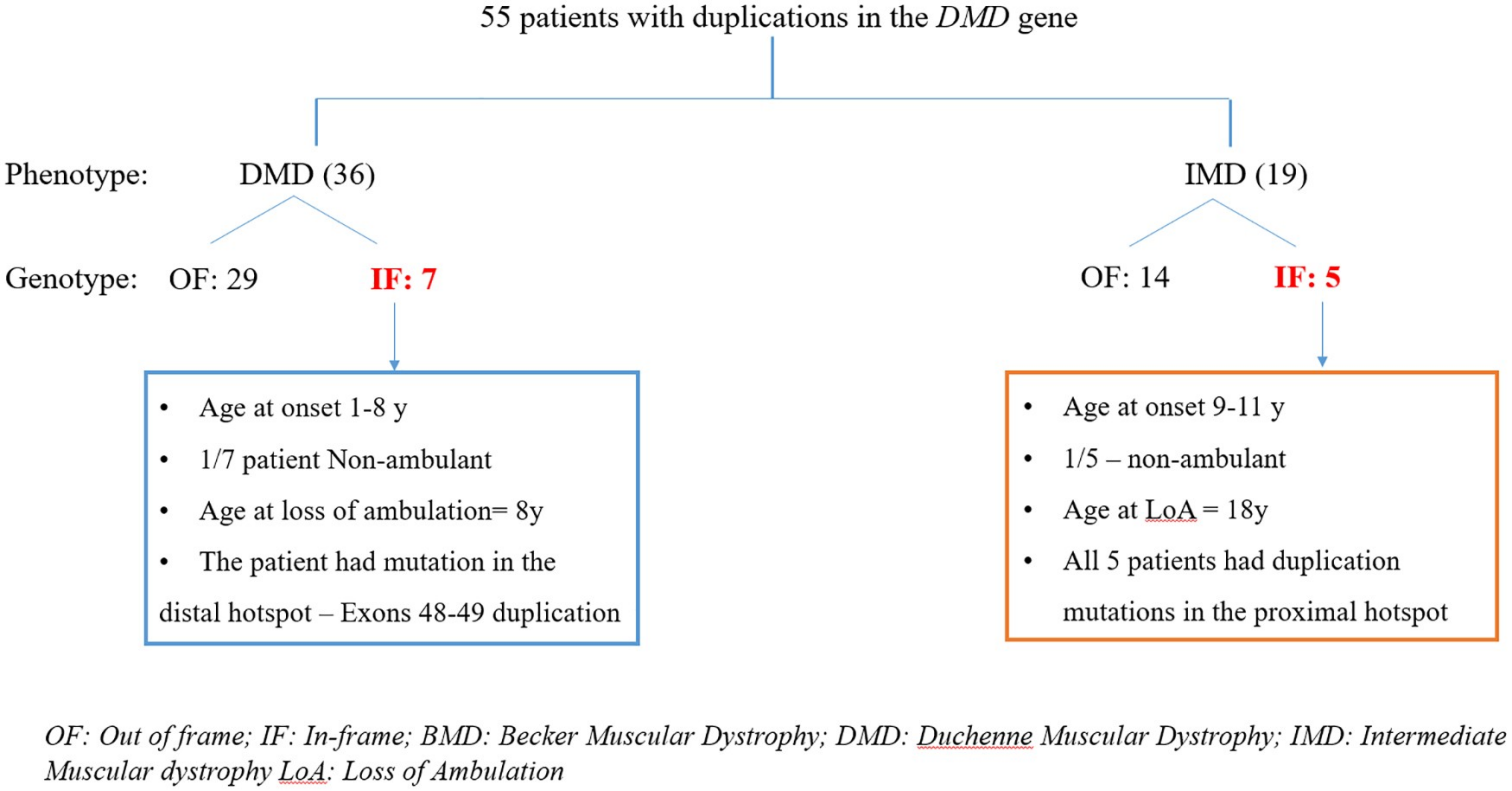
Genotype: Phenotype overview of patients with duplication mutations in the *DMD* gene. The number of patients in each segment has been numerically indicated. The number of patients defying the reading frame rule is marked in red. OF: out of frame; IF In-frame; BMD: Becker’s Muscular Dystrophy; IMD: Intermediate Muscular Dystrophy; DMD: Duchenne Muscular Dystrophy; LoA: Loss of ambulation.

Analysis of 55 patients with duplications in the *DMD* gene for whom clinical data on age at onset of disease and age at loss of loss of ambulation were available, was next performed. No patients with a diagnosis of BMD were identified in this cohort (Fig 5).

Of the 36 clinically diagnosed DMD patients, 29 were found to have out of frame mutations while 7 had in-frame mutations. The age of onset of symptoms in these 7 patients ranged from 1–8 years. Only one patient had lost ambulation at 8 years of age. Of 19 IMD patients, 14 were found to have out of frame mutations, while 5 were identified with in-frame mutations in the proximal hotspot region of the *DMD* gene. One of these patients was found to have lost ambulation at the age of 18 years (Fig 5).

### A review of in-frame mutations in DMD patients revealed an enrichment of mutations originating in the proximal hotspot region

**Fig 6:**
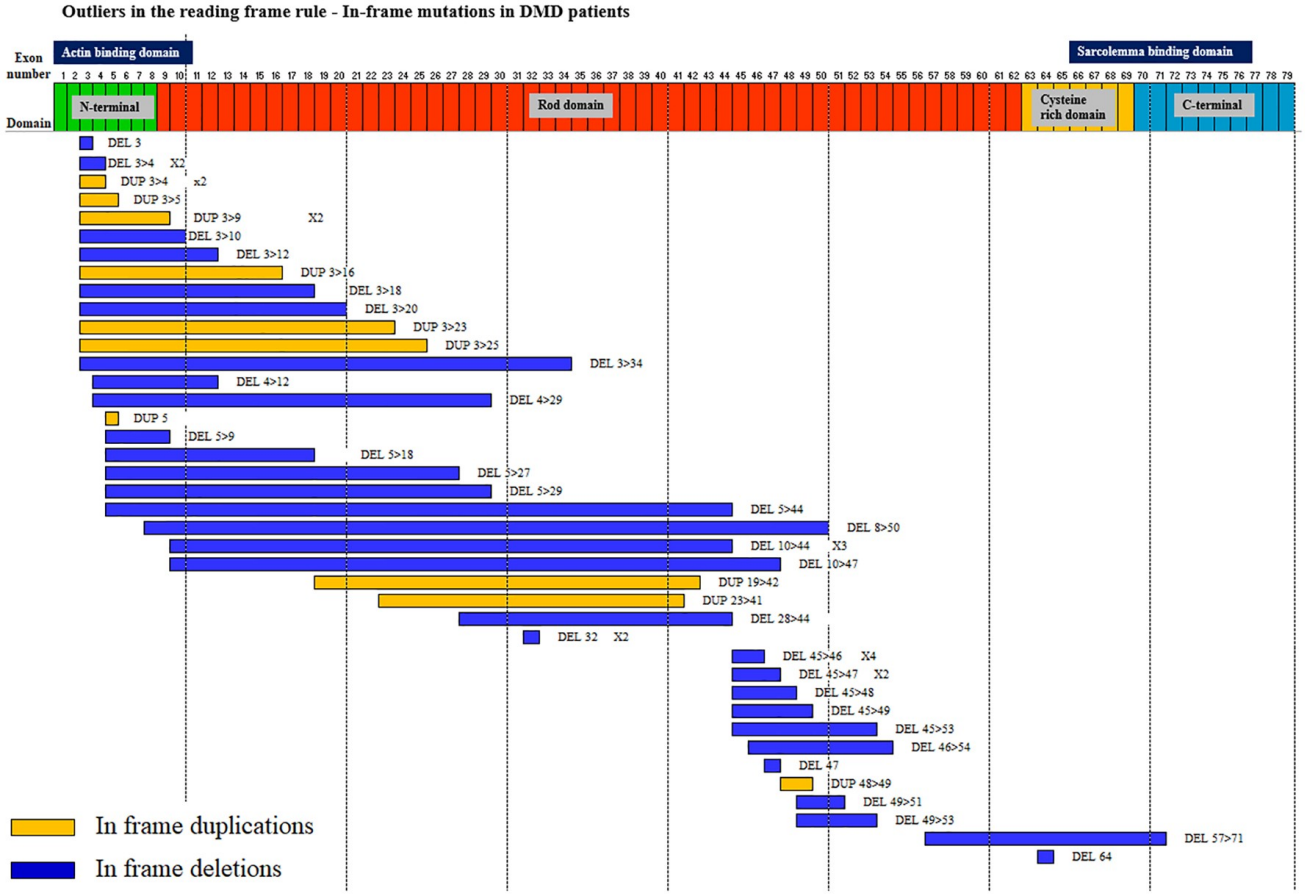
A summary of in-frame mutations in DMD patients shows an enrichment of mutations arising in the proximal *DMD* gene hotspot. 50 in-frame deletion and duplication mutations in DMD patients were mapped to the 79 exons of the *DMD* gene, which is depicted with its functional domains: N-terminus, Rod domains, Cysteine rich domain and C-terminus. The precise mutation in each case and its frequency (where applicable) has been indicated. In-frame duplications are in yellow. In-frame deletions are in blue.

Based on our observations of deletion and duplication mutations in the *DMD* gene in DMD patients, we surmised that patients with in-frame mutations who defied the reading frame rule, could not be clinically distinguished from DMD patients harboring out of frame mutations. In order to determine if DMD patients with in frame mutations had common genotypic features, we mapped the deletions and duplications identified in this cohort within the 79 exons of the *DMD* gene (Fig 6).

The majority of mutations (60%) had their origins in the proximal hotspot region (exons1-22) of the *DMD* gene. This is in contrast to of out-of-frame *DMD* mutations, the majority (90%) of which fall in the distal hotspot region of the *DMD* gene (Fig 3).

### Next Generation Sequence (NGS) analysis revealed DMD patients with small mutations

The remaining 246 patients (25.6%) in our cohort of 961 patients, (Fig 1), were subjected to NGS, which revealed 101 patients with small deletions/ duplications or point mutations in the *DMD* gene. These mutations were undetectable by mPCR and MLPA. Further analysis of the 101 patients revealed 90 with predicted pathogenic mutations, 4 with likely pathogenic mutations and 7 with mutations (variants) of unknown significance (S3 Table). NGS analysis further revealed that one third of DMD patients harbored nonsense mutations (S2 Fig) and C>T transitions appeared to be favored in these patients (S3 Fig). Collectively, NGS analysis added to the number of patients with *DMD* gene mutations, increasing the diagnosis of DMD from 74.5% (mPCR and MLPA) to a total of 84.9% patients with DMD or BMD in our cohort.

No *DMD* gene mutation was found in 145 patients despite being clinically suspected of having DMD. The majority of these patients (Fig 1; 100) were found to have Limb Girdle Muscular Dystrophies (LGMD), Bethlem myopathy and Emery Driefus myopathy (S4 Fig), the details of which will be reported elsewhere (manuscript in preparation). 29 patients were found to have other disorders with some overlapping clinical symptoms with DMD. These included patients diagnosed with Axonal Charcot-Marie Tooth syndrome, Nemaline myopathy, among others.

NGS analysis revealed 16 patients in our cohort with no mutation found in the genes interrogated in our gene panel (S2 Table). In all likelihood these patients did not have muscular dystrophies, but had other diseases that could have been identified had our NGS analysis been expanded to whole exome or even whole genome sequencing.

### Identification of rare genetic mutations in the *DMD* gene revealed

Analysis of all *DMD* gene mutations in our cohort of 961 suspected DMD patients, identified a number of novel mutations. These included 40 novel deletions, 16 novel duplications and 51 novel point mutations (Table 1, and S4A, S4B, S4C and S5 Tables). Additionally, 9 non-contiguous mutations were identified. Details of all novel mutations in the *DMD* gene were deposited in the Leiden Open Variation Database (LOVD) with a unique identifier number assigned to each mutant (S4A, S4B, S4C and S5 Tables).

Interestingly, 90% of DMD patients with novel deletion mutations had their exon start points in the proximal hotspot region (exons 1–22) of the *DMD* gene (Figs 3 and 6), with 33% of patients with deletions originating in exon 8 and 23% of patients with deletions beginning in exon 3. Both exon 3 (37% DMD patients) and exon 8 (25% of DMD patients) were also featured prominently in the novel duplications we identified (S4A and S4B Table). The distribution of novel point mutations in the *DMD* gene on the other hand, (40 exonic and 11 intronic mutations), appeared to cluster in the distal region of the *DMD* gene (S5 Table).

**Fig 7:**
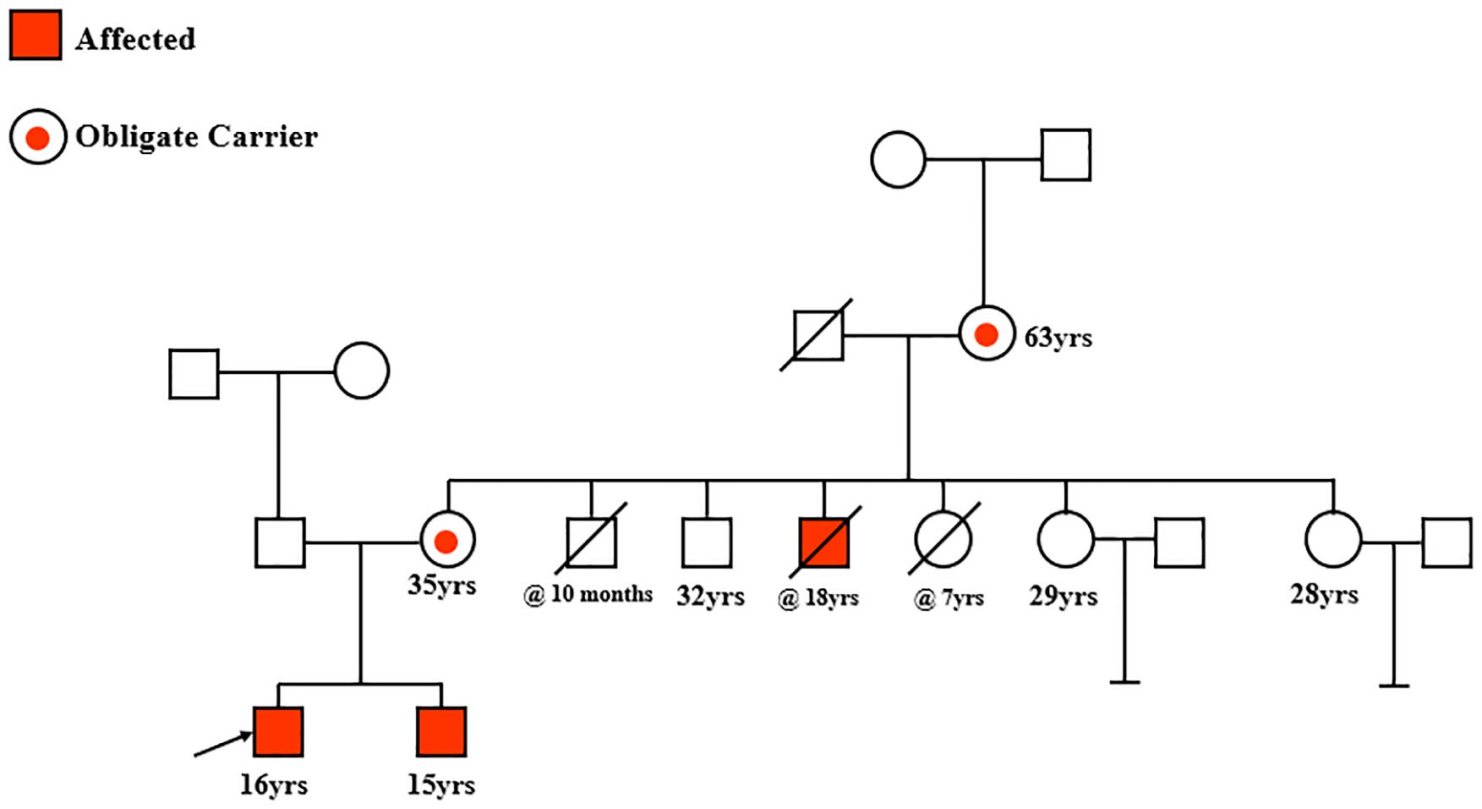
Pedigree of a family with a rare non-contiguous deletion mutation in the *DMD* gene: Del exons 45–50 and 53–54.

Among the novel non-contiguous deletion mutations, we identified a rare mutation involving Exon 45–50 and 53–54 (Fig 7, Table 4 and S4C Table).

The proband (marked with black arrow), his brother and a deceased uncle were found to have this rare non-contiguous mutation. Two carrier females, the mother and grandmother (circles with red dot), also harbored this rare non-contiguous mutation.

A rare noncontiguous *DMD* gene mutation involving deletions in exons 45–50 and 53–54 was identified in a 16 year old patient diagnosed with DMD (Fig 7, proband; black arrow) Upon further interrogation on his family history of disease, his brother aged 15years was also found to be symptomatic and to harbor the same mutation. An uncle of the two affected brothers had previously been shown to have this mutation, but had since expired. The pedigree illustrated in Fig 7 shows the three male members affected with DMD. Given that three closely related males all harbored the same rare *DMD* gene deletion mutation, prompted us to determine the carrier status of the mother and grandmother of the two affected brothers, for whom blood samples were available. Interestingly, both the mother of the affected proband and his grandmother (circles with red dot), were found to be carriers of this mutation. A 32 year old uncle of the affected brothers was found to lack this mutation and remains disease-free. The carrier status of two of their aunts aged 29 and 28 years remains undetermined thus far. Our data suggest that this rare non-contiguous *DMD* gene mutation is causative of familial DMD in affected males and could be traced to carrier females in this pedigree.

### Analysis of mutations in the *DMD* gene reveal opportunities for treatment of a subset of DMD patients

In recent times, newer innovative small molecule therapeutic approaches to treat DMD have emerged and include exon skipping and stop codon read through. The basic premise of exon skipping has been to transform out- of- frame to in-frame mRNAs leading to the production of truncated, but partially functional dystrophin protein, thereby deferring disease progression [24,25]. A survey of confirmed DMD patients in our cohort identified patients with deletion mutations who are likely to benefit from an exon skipping strategy for therapy (Table 2).

**Table 2:** Overview of deletions mutations for which single exon skipping is applicable.

S.No	Exon amenable for skipping	No.of patients eligible	% total mutation	% deletion
1	51	117	14.3	18.1
2	53	88	10.7	13.6
3	46	41	5.0	6.3
4	55	40	4.9	6.2
5	45	26	3.2	4.0
6	52	18	2.2	2.8
7	44	15	1.8	2.3
8	8	7	0.9	1.1
9	43	0	0	0
10	50	0	0	0
**TOTAL**		**352**		

Total number of patients eligible for multiple exon skipping (Exons 45–55) = **465/642 deletions**

The majority of patients (117;14.3%) were predicted to benefit from exon 51 skipping, followed by exon 53 and exon 46 skipping. This finding is of significance, given that two exon skipping drugs Exondys 51 (exon 51 skipping) and Golodirsen (exon 53 skipping), have recently been approved by the FDA. While the former has shown a very modest increase in dystrophin levels, no measurable functional improvements have been shown yet. Additionally, a total of 465 of 642 DMD patients with deletions mutations were predicted to benefit from a multiple exon skipping (exons 45–55) approach, as recently suggested by the Yokota group [26].

Furthermore, 35 patients were identified with nonsense mutations leading to stop codons (Table 3).

**Table 3:** Patients with nonsense mutations in the *DMD* gene.

S.No	Patient ID	Age at diagnosis (Years)	Age at onset of symptoms	Ambulation status at the time of diagnosis	Age at loss of Ambulation	Exon	Variant identified	Amino acid change
						Location		
1	0025/B-23/072006	3	2	Ambulant	Not applicable	Exon 34	c.4729C>T	Arg1577Ter
2	0051/B-49/012007	8	Not Available	Not Available	Not Available	Exon 14	c.1615C>T	Arg539Ter
3	0089/B-97/042007	4	3	Ambulant	Not applicable	Exon 23	c.3087G>A	Trp1029Ter
4	0110/B-122/062007	9	7	Ambulant	Not applicable	Exon 48	c.6955C>T	Gln2319Ter
5	0122/B-134/072007	11	5	Ambulant	Not applicable	Exon 21	c.2776C>T	Gln926Ter
6	0128/B-140/072007	9	7	Ambulant	Not applicable	Exon 20	c.2582C>G	Ser861Ter
7	0147/B-154/092007	7	4	Ambulant	Not applicable	Exon 6	c.397C>T	Gln133Ter
8	0163/B-168/102007	6	5	Ambulant	Not applicable	Exon 44	c.6428G>A	Trp2143Ter
9	0207/B-218/022008	9	1	Ambulant	Not applicable	Exon 15	c.1721G>A	Trp574Ter
10	0213/B-224/022008	8	3	Ambulant	Not applicable	Exon 66	c.9568C>T	Arg3190Ter
11	0256/B-293/052008	8	8	Ambulant	Not applicable	Exon 37	c.5255T>G	Leu1752Ter
12	0273/B-320/072008	2	2	Ambulant	Not applicable	Exon 48	c.6979G>T	Glu2327Ter
13	0301/B-366/102008	18	16	Ambulant	Not applicable	Exon 35	c.4948G>T	Glu1650Ter
14	0652/B-773/062009	8	7	Ambulant	Not applicable	Exon 6	c.457C>T	Gln153Ter
15	0691/DBI-599/092009	8	8	Ambulant	Not applicable	Exon 21	c.2642C>G	Ser881Ter
16	0773/DBI-682/032010	7	4	Ambulant	Not applicable	Exon 34	c.4729C>T	Arg1577Ter
17	0775/DBI-684/042010	7	6 months	Ambulant	Not applicable	Exon 64	c.9337C>T	Arg3113Ter
18	0910/DBI-783/072010	6	5	Ambulant	Not applicable	Exon 17	c.2047G>T	Glu683Ter
19	0966/DBI-832/102010	12	3	Non Ambulant	7	Exon 69	c.10033C>T	Arg3345Ter
20	1006/DBI-858/112010	5	5	Ambulant	Not applicable	Exon 60	c.8944C>T	Arg2982Ter
21	1052/DBI-889/122010	10	7	Non Ambulant	10	Exon 26	c.3595G>T	Glu1199Ter
22	1202/DBI-1012/052011	6	6	Ambulant	Not applicable	Exon 23	c.2968C>T	Gln990Ter
23	1261/DBI-1050/072011	5	2	Ambulant	Not applicable	Exon 57	c.8420G>A	Trp2807Ter
24	1270/DBI-1058/072011	9	7	Ambulant	Not applicable	Exon 6	c.394C>T	Gln132Ter
25	1278/DBI-1067/072011	6	6	Ambulant	Not applicable	Exon 19	c.2302C>T	Arg768Ter
26	1331/DBI-1109/082011	10	7	Ambulant	Not applicable	Exon 55	c.8038C>T	Arg2680Ter
27	1349/DBI-1129/082011	8	2	Ambulant	Not applicable	Exon 24	c.3224G>A	Trp1075Ter
28	1407/DBI-1173/112011	8	4	Ambulant	Not applicable	Exon 29	c.3923C>G	Ser1308Ter
29	1459/DBI-1214/012012	8	7	Ambulant	Not applicable	Exon 39	c.5542A>T	Lys1848Ter
30	1607/DBI-1319/042012	8	4	Ambulant	Not applicable	Exon 41	c.5899C>T	Arg1967Ter
31	1685/DBI-1378/062012	21	Not Available	Non Ambulant	14	Exon 70	c.10219G>T	Glu3407Ter
32	1728/DBI-1411/072012	8	8	Ambulant	Not applicable	Exon 14	c.1702C>T	Gln568Ter
33	1940/DBI-1557/112012	9	5	Non Ambulant	9	Exon 44	c.6407G>A	Trp2136Ter
34	1946/DBI-1560/122012	12	4	Non Ambulant	11	Exon 41	c.5899C>T	Arg1967Ter
35	2198/DBI-1754/032013	13	9	Ambulant	Not applicable	Exon 53	c.7792C>T	Gln2598Ter

These patients could potentially benefit from the small molecules recently approved in Europe that are involved in stop codon read through, leading to rescue of the *DMD* mRNA, thereby reestablishing dystrophin protein expression.

It has been previously shown that non-contiguous mutations in the *DMD* gene often escape the “reading frame rule” resulting in intermediate phenotypes and account for 1% of *DMD* gene mutations [27]. In our cohort of 961 suspected DMD patients, we identified 9 patients with non-contiguous mutations (Table 4).

**Table 4:** Non-contiguous mutations in the *DMD* gene.

S.No	Patient ID	Age of patient at diagnosis (Years)	Age at onset of symptoms	Ambulation status—at the time of diagnosis	Age at loss of Ambulation	Mutation (mPCR/MLPA)	Amenable for multiple exon skipping (Exons 45–55)
1	0103/B-110/052007	8	8	Ambulant	Not applicable	Exons 45–50 & 53–54 Deletion	Amenable
2	0176/B-181/112007	9	4	Ambulant	Not applicable	Exons 45–48 & 53–55 Duplicated	Not amenable
3	0190/B-198/122007	7	6	Ambulant	Not applicable	Exons 3–9 & 18–44 Duplicated	Not amenable
4	0227/B-244/042008	7	3	Ambulant	Not applicable	Exons 52–62 & 66–79 Duplicated	Not amenable
5	4772/DBI-3603/032018	6	5	Ambulant	Not applicable	Exons 3–10 & 18–41 Deletion	Not amenable
6	0896/DBI-775/072010	13	7	Non Ambulant	7	Exons 17–42 & 45–52 Duplicated	Not amenable
7	2450/DBI-2004/102013	9	2.5	Ambulant	Not applicable	Exons 45–55 & 63 Duplicated	Not amenable
8	0974/DBI-840/102010	10	5	Non Ambulant	9	Exon 5 Deletion & 6 Duplication	Not amenable
9	1719/DBI-1402/072012	9	8	Ambulant	Not applicable	Exons 48, 51–55 Duplication	Not amenable

Based on deletions or duplications of exons in the *DMD* gene in each patient, we predicted that non-contiguous mutations led to out-of frame mutations in 7 of 9 patients (Table 4). However, 2 of 9 patients, despite having non-contigous mutations are likely to be amenable to an exon skipping strategy involving exons 45–55 [26]. Should such a strategy become therapeutically viable in the future, these two patients, including one with the same mutaion described in the pedigree above (Fig 7), could benefit, presumably through the expression of a shorter mRNA leading to a truncated, but probably functional DMD protein.

### Identification of the carrier status of mothers of DMD probands provides opportunities for prevention of DMD

Having identified 816 probands with mutations in the *DMD* gene with a confirmed diagnosis of DMD, we sought to determine the carrier status of mothers and female relatives of these patients. 463 female relatives consented to provide carrier information and provided blood samples for DNA analysis which was collected during proband counseling sessions. As is indicated in Table 5, 316 mothers and 147 other female relatives of probands with known *DMD* gene mutations were tested for the same mutations in the *DMD* gene.

**Table 5:** An assessment of the carrier status of female relatives of 816 DMD patients.

Total no.of confirmed DMD probands = **816**				
No.of female relatives from mutation positive probands who have consented to provide sample for carrier analysis = **463**				
**Numbers**	**Mother**	**Sister**	**Daughter**	**Other female relatives**
Total	316	125	2	20
Carrier	138 (44%)	26 (21%)	2 (100%)	4 (40%)
Normal	178	99	0	16
**Type of mutation**	**No.of samples received for carrier analysis**			**No.of carrier positives**
Deletion	404			137
Duplication	42			19
Small mutation	17			14

**Fig 8:**
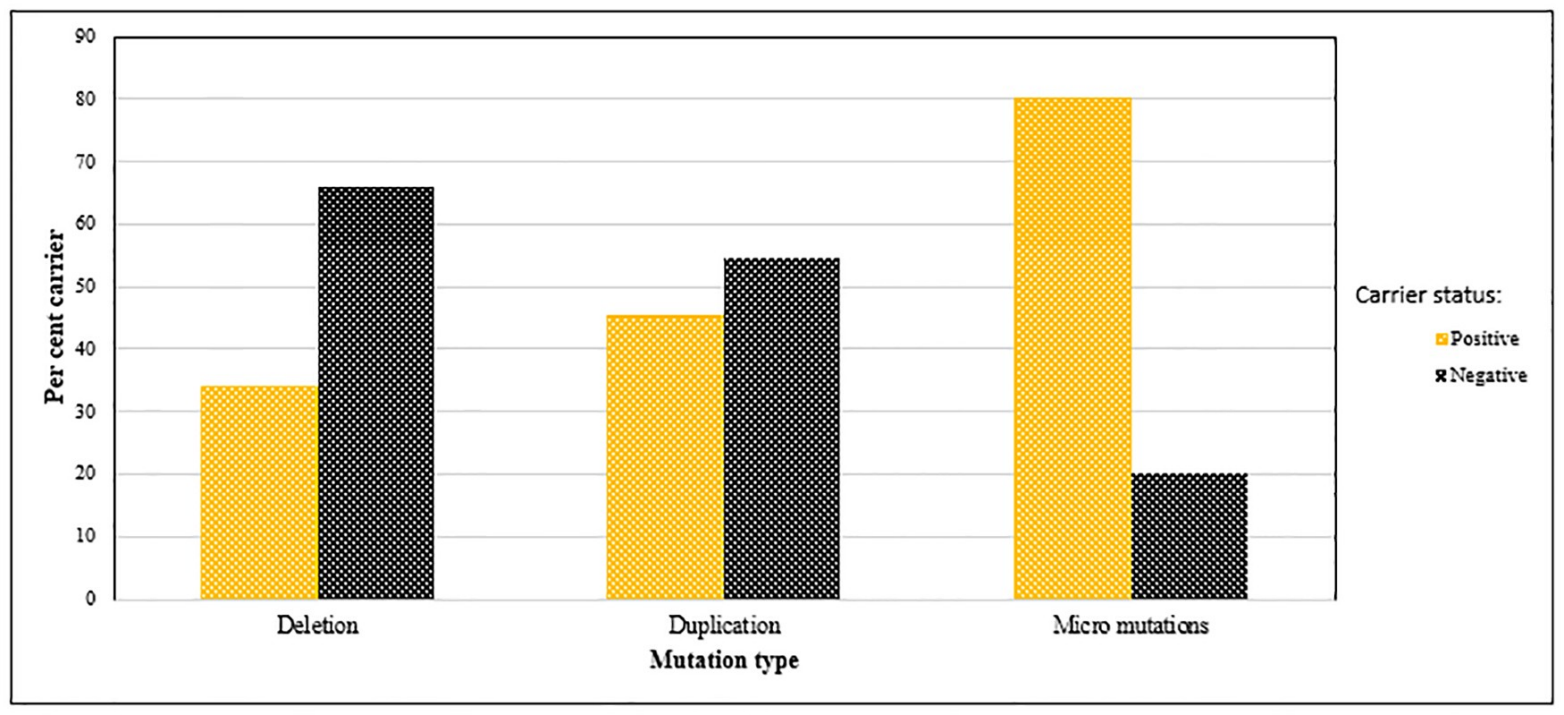
Carrier frequencies based on type of mutations in the *DMD* gene. Deletions, duplications and micro (point) mutations occurring in both carrier (positive) and normal (negative) mothers was assessed. Point mutations were most frequent in carrier mothers.

Carriers with mutations in the *DMD* gene were identified as a function of the total proband numbers with a particular class of mutation. Thus, (Table 5), 137/404, (33.9%) carriers were deletion mutation positive, 19/42 (45.3%) had duplications and 14/17 (80%) harbored small mutations. As graphed in Fig 8, carriers appear to favor point mutations, even though the total number of such mutations is comparatively low.

Based on our analysis, an estimate of the numbers of inherited versus sporadic cases of DMD could be calculated. In contrast to the theoretically calculated estimate of inherited DMD of two thirds in a given cohort examined [28,29], our observation appeared to be significantly lower at 43.7% (138/316) with sporadic cases estimated at 56.3%.

## Discussion

We present here the first large comprehensive genetic study on DMD patients from India. Previous studies from India aiming to diagnose DMD at the genetic level have thus far used mPCR and MLPA methods [30–33]. In one study, the DMD gene mutation detection rate was reported to be 62% leaving 36% of clinically suspected DMD patients with no detected mutations [33], underscoring the need for a comprehensive diagnostic approach. More recently a small study conducted in India focused on NGS as a single platform replacing the need for mPCR and MLPA in diagnosing patients with DMD [34]. However, larger numbers of patients need to be considered before the NGS platform alone can be confirmed as a single comprehensive platform for the diagnosis of DMD.

In light of this, we implemented a simple, minimally invasive algorithm which includes NGS, particularly suited for developing and low income countries, and used this approach to diagnose DMD or BMD in a cohort of 961 clinically suspected DMD patients. In the process, we have made several significant observations with important repercussions for DMD patients. Given that an accurate diagnosis of DMD is an essential first step to implementing an effective disease management strategy [35], we first used multiplex PCR as an inexpensive and easy method to initially identify common hot spot deletion mutations in 30 of the 79 exons in the *DMD* gene. Patients lacking such mutations, and those in which the borders of deletion mutations were unclear, were then tested by MLPA, which is designed to identify large deletions and duplications in the *DMD* gene. While the majority of patients (75%) tested positive for *DMD* gene mutations by these two methods, 25% of patients remained undiagnosed and hence were tested by the relatively more expensive NGS method, yielding an additional 10% of patients with “small” mutations in the *DMD* gene. NGS analysis revealed that C>T transition followed by G>A transitions were the most common point mutations in our cohort, and invariably resulted in termination mutations. While all C>T transitions resulted in termination mutations, all termination mutations did not harbor C>T transitions. This observation has been previously reported in a study where a C>T transition in a hotspot CpG dinucleotide island region in the *DMD* gene (C.8713C>T/p.R2905X) was observed [36]. The reason for the large numbers of C>T transitions in DMD associated point mutations remains unclear.

The majority of the remaining 15% of patients who lacked *DMD* gene mutations were found to harbor mutations in genes associated with other muscular dystrophies (OMDs), particularly Limb Girdle Muscular Dystrophies (LGMDs), or other unrelated disorders such as Charcot Marie Tooth and Nemaline myopathy. This set of patients would have most likely been misdiagnosed as having DMD, a disease with considerably higher morbidity and mortality compared to other MDs, without the NGS component of our diagnostic algorithm. NGS is fast replacing other methods of genetic diagnosis, particularly since the price tag associated with it has plummeted over the past decade, making it relatively more accessible to patients, especially in developed countries. Our algorithm is recommended particularly in communities, such as in rural Tamil Nadu, India, where the cost of diagnostic tests could decide whether a patient undergoes the test or not. Thus, using the NGS option as a last step in our algorithm has made DMD diagnosis both accurate and affordable for most patients. The inclusion of NGS has also circumvented the need for painful muscle biopsies [37], which were previously required on a routine basis in the event the diagnostic results from mPCR or MLPA tests were found to be negative [32,38].

Ninety eight percent of the 961 clinically suspected DMD patients in our cohort received a definite genetic diagnosis, lending credence to the significance of our diagnostic algorithm. Sixteen patients from our cohort of 961 (2%) were however, found to be devoid of mutations in genes interrogated in our NGS gene panel, despite having some DMD-like clinical manifestations. Further genetic clarity on these patients will likely emerge upon whole exome or whole genome sequencing of their blood or muscle biopsy samples.

The identification of 50 DMD patients with in-frame deletions or duplications in the *DMD* gene of which 30 (60%) had mutations originating in the proximal hotspot region of the *DMD* gene was surprising, because a recent study reporting 24 in-frame DMD patients showed a preference for mutations in the distal hotspot region of the *DMD* gene [39]. Similar analysis on larger numbers of such patients will help resolve this apparent inconsistency. Our finding that in-frame mutations in DMD patients are associated with a severe phenotype could be explained by the enrichment of mutations in this cohort in the proximal hotspot region, which more than likely results in the abrogation of *DMD* mRNA expression and loss of the DMD protein.

According to the TREAT-NMD DMD database, approximately 10–15% of DMD patients have a nonsense mutation in the DMD gene [21], leading to protein truncation and subsequent degradation. The finding that aminoglycoside antibiotics such as Gentamycin can interact with ribosomes and allow for stop codon read through, resulting in the formation of a full length protein [40], led to the development of Ataluren (Translarna©) (PTC Therapeutics, USA). This drug had previously been shown to restore levels of the dystrophin protein in cell lines and in a mouse model of DMD (mdx mice). However, in the phase 2b randomized, double-blind, placebo controlled clinical trial (PTC124-GD-007-DMD), a small but measurale beneficial effect of ataluren was accepted to be clinically relevant, and provided a much needed ray of hope for DMD patients. Based on this, conditional approval of Atalauren for use in Europe was granted in 2014 by the European Commission [41]. We identified 35 patients in our cohort of 101 NGS tested patients harboring a stop codon, who might potentially benefit from Ataluren treatment.

Another mutation specific therapeutic option involving exon skipping involves the use of antisense oligomers (AOs) designed to target specific exons in the *DMD* gene. Success using this approach has led to FDA approval of Exondys51 and Golodirsen (Sarepta, USA), antisense drugs specific for exon 51 and exon 53 skipping respectively, which applies to the largest group of patients [42]. Similar AOs are also being developed for exon 45. A survey of our DMD cohort also found the largest number of patients (117) amenable to exon 51 skipping. These patients could potentially benefit from exon skipping therapy, which is currently undergoing further refinement in AO chemistry to improve uptake by muscle cells [42].

More recently, the therapeutic rationale for skipping Exons 45–55 has been proposed by Toshifumi Yokota [22] and is based on the well documented observation that patients with in-frame deletion mutations in the *DMD* gene involving exons 45–55 deletions result in mild symptoms or may even be asymptomatic [26,43]. It is presumed that mRNA stability following exon 45–55 deletion likely contributes to this phenomenon. Our data are consistent with this observation, as 12/14 patients diagnosed with DMD having in- frame deletion mutations involving exons 45–55 were ambulant, with two in particular, who despite having onset of symptoms at ages 5 and 10 remained ambulant at ages 27 and 22 respectively. Our data also show that 14 BMD patients with deleted exons 45–55 had mild symptoms with an average age of onset at 15.2 years and age of diagnosis at 27.6 years. All 14 BMD patients remained ambulant.

We have further identified *DMD* gene mutations in 138 /316 consenting carrier mothers of DMD probands, demonstrating that 43.7% of DMD cases analyzed in our cohort had familial disease while the rest were sporadic. This percentage is significantly lower than the theoretically derived value of two thirds of carrier mothers being predicted to be carrier positive [28,29]. This discrepancy requires further insight and study in larger cohorts of patients, specifically in the large DMD databases.

Our study has demonstrated that the power of mutational analysis coupled with genetic counseling can provide DMD carrier mothers with the opportunity to make informed family planning decisions to prevent the birth of subsequent DMD positive offspring. This is of vital importance particularly in rural India where impoverished mothers, often alone, are left to shoulder the burden of care for, in some cases, multiple DMD afflicted sons. In lieu of currently available effective treatment options for patients with DMD, and with the multi-exon skipping approach likely to face regulatory challenges and unlikely to be developed clinically in the near future [35], informed family planning decisions hold great promise to prevent the propagation of DMD, an invariably deadly disease.

## Supporting information

S1 Fig Overview of the mutational spectrum in 961 patients. (PPTX)

S2 Fig Types of mutations found in 94 DMD patients analysed by NGS. (PPTX)

S3 Fig Types of nucleotide transitions observed in 92 DMD patients analyzed by NGS. (PPTX)

S4 Fig Other muscular dystrophies identified. (PPTX)

S1 Table Clinical symptoms of 961 suspected DMD patients. (PPTX)

S2 Table Muscular dystrophy and congenital myopathy gene panel for NGS analysis. (PPTX)

S3 Table Variants of uncertain significance in the *DMD* gene. (PPTX)

S4 Table Novel *DMD* gene mutations identified in our cohort of 961 suspected DMD patients with LOVD submission numbers A: 40 novel deletions B: 16 novel duplications C: 8 novel non-contiguous mutations. (PPTX)

S5 Table 51 Novel *DMD* point mutations identified in our cohort of 961 suspected DMD patients with LOVD submission numbers. (PPTX)
