# Supplementary material for: Comprehensive genetic analysis of 961 unrelated Duchenne Muscular Dystrophy patients: Focus on diagnosis, prevention and therapeutic possibilities
Source: PLoS One. 2020 Jun 19;15(6):e0232654. doi: 10.1371/journal.pone.0232654 (PMC7304910; doi:10.1371/journal.pone.0232654)
Supplement: S1 Fig — (PPTX) [file pone.0232654.s001.pptx]

## Slide 1
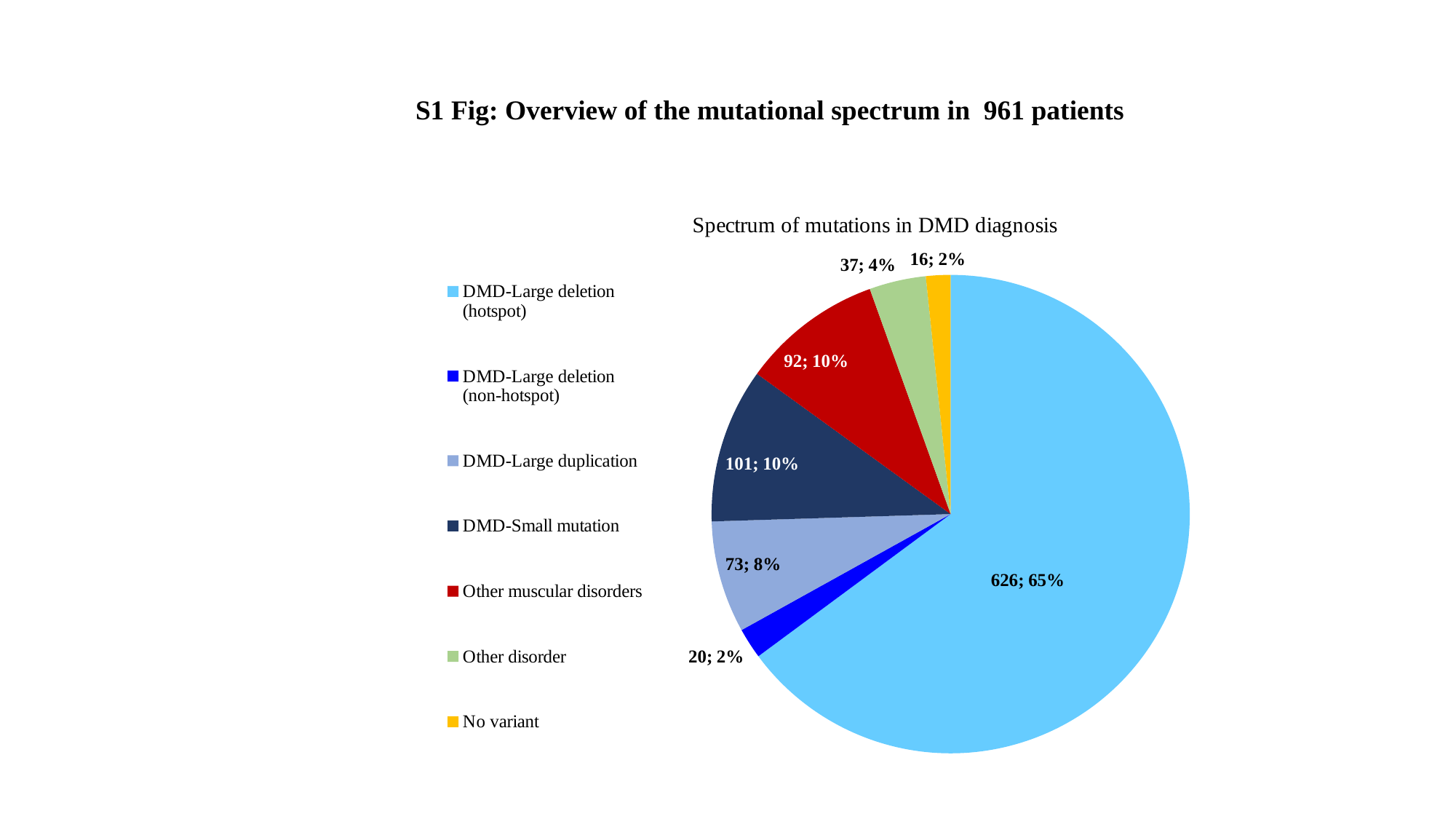

S1 Fig: Overview of the mutational spectrum in 961 patients
### Chart: Spectrum of mutations in DMD diagnosis
| Category | |
|---|---|
| DMD-Large deletion (hotspot) | 626.0 |
| DMD-Large deletion (non-hotspot) | 20.0 |
| DMD-Large duplication | 73.0 |
| DMD-Small mutation | 101.0 |
| Other muscular disorders | 92.0 |
| Other disorder | 37.0 |
| No variant | 16.0 |
