# Supplementary material for: Comprehensive genetic analysis of 961 unrelated Duchenne Muscular Dystrophy patients: Focus on diagnosis, prevention and therapeutic possibilities
Source: PLoS One. 2020 Jun 19;15(6):e0232654. doi: 10.1371/journal.pone.0232654 (PMC7304910; doi:10.1371/journal.pone.0232654)
Supplement: S2 Fig — (PPTX) [file pone.0232654.s002.pptx]

## Slide 1
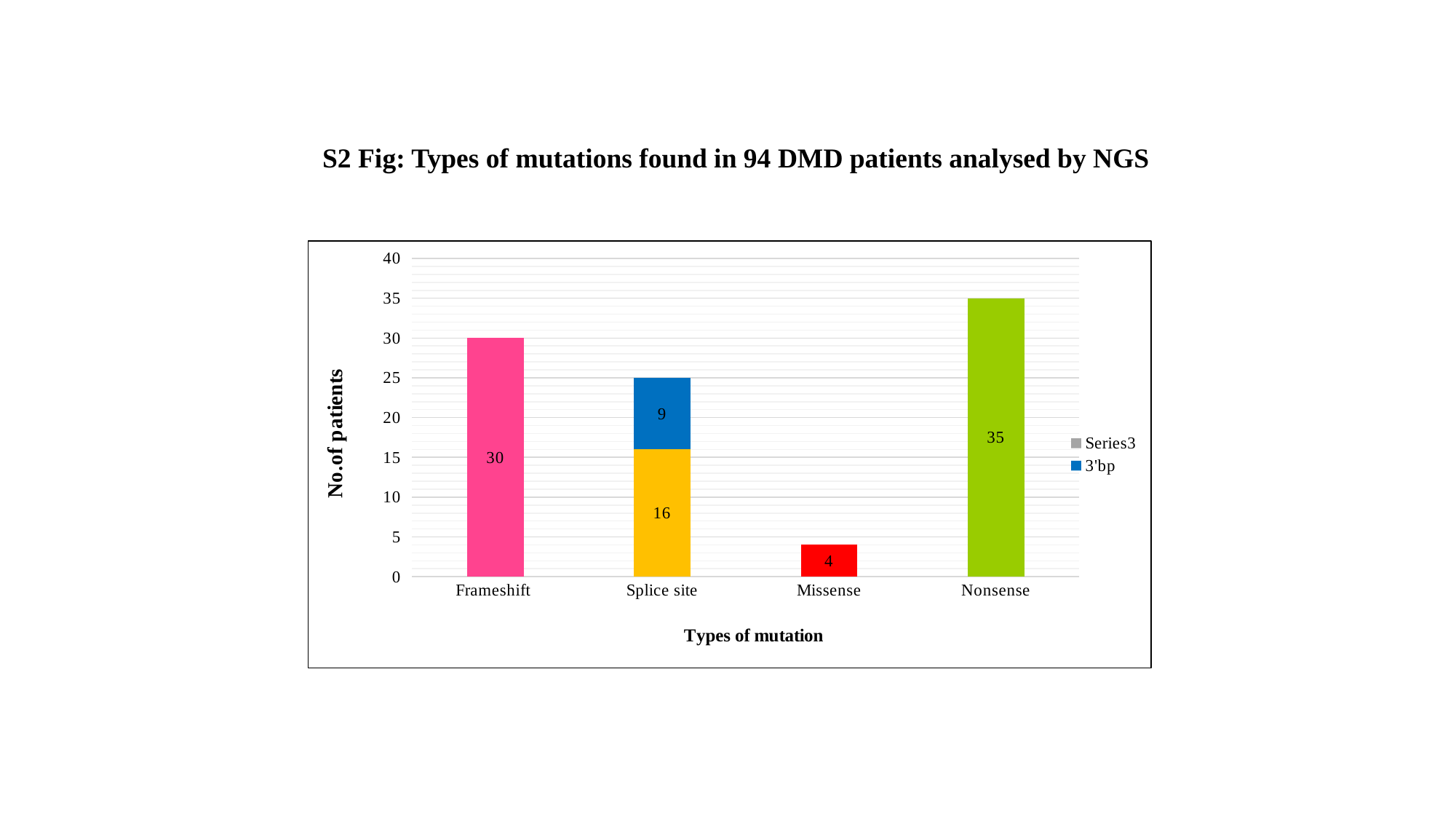

S2 Fig: Types of mutations found in 94 DMD patients analysed by NGS
### Chart
| Category | 5'bp | 3'bp | |
|---|---|---|---|
| Frameshift | None | None | 30.0 |
| Splice site | 16.0 | 9.0 | None |
| Missense | None | None | 4.0 |
| Nonsense | None | None | 35.0 |
