# Supplementary material for: Comprehensive genetic analysis of 961 unrelated Duchenne Muscular Dystrophy patients: Focus on diagnosis, prevention and therapeutic possibilities
Source: PLoS One. 2020 Jun 19;15(6):e0232654. doi: 10.1371/journal.pone.0232654 (PMC7304910; doi:10.1371/journal.pone.0232654)
Supplement: S3 Fig — (PPTX) [file pone.0232654.s003.pptx]

## Slide 1
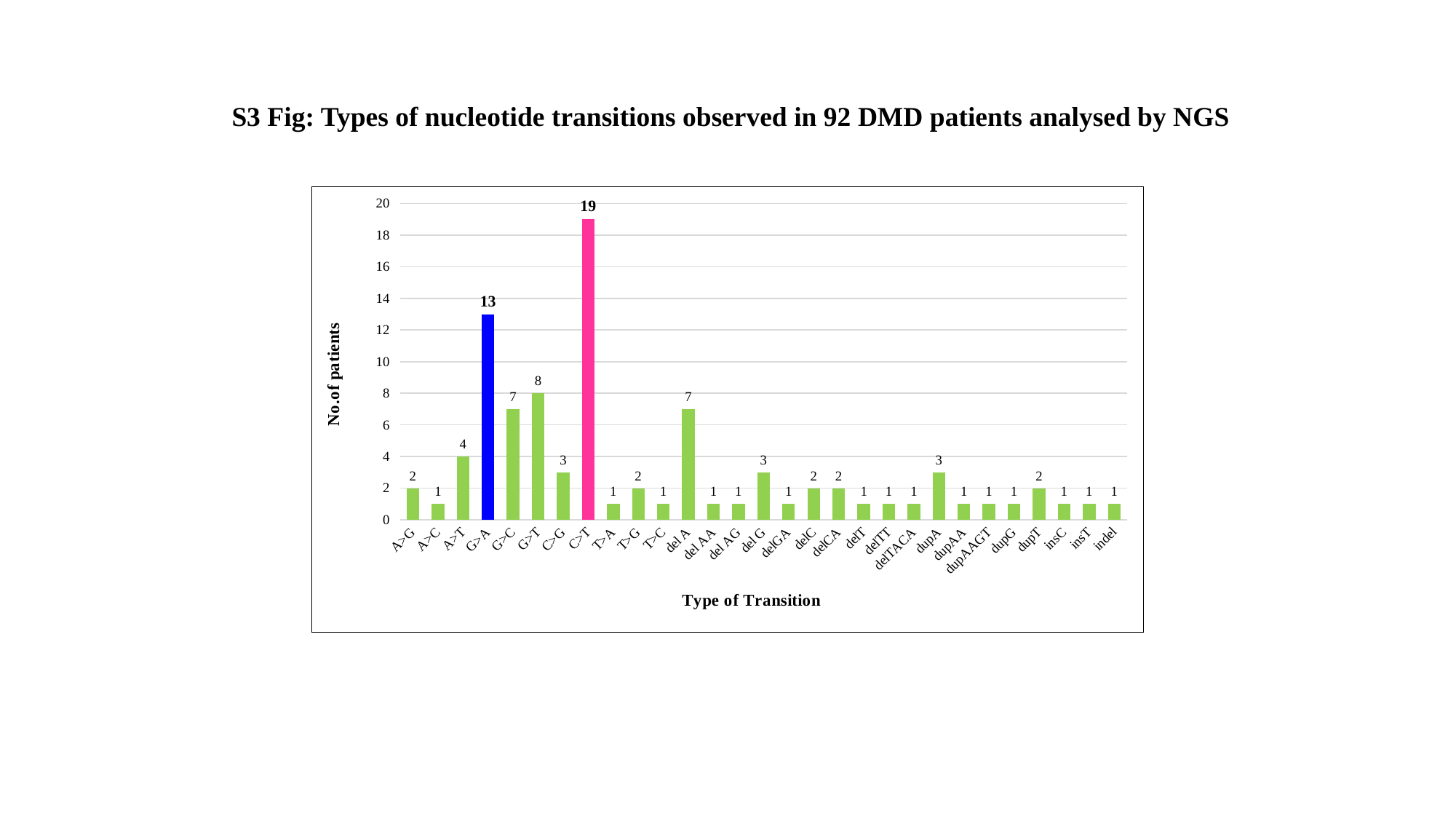

S3 Fig: Types of nucleotide transitions observed in 92 DMD patients analysed by NGS
### Chart
| Category | No.of patients |
|---|---|
| A>G | 2.0 |
| A>C | 1.0 |
| A>T | 4.0 |
| G>A | 13.0 |
| G>C | 7.0 |
| G>T | 8.0 |
| C>G | 3.0 |
| C>T | 19.0 |
| T>A | 1.0 |
| T>G | 2.0 |
| T>C | 1.0 |
| del A | 7.0 |
| del AA | 1.0 |
| del AG | 1.0 |
| del G | 3.0 |
| delGA | 1.0 |
| delC | 2.0 |
| delCA | 2.0 |
| delT | 1.0 |
| delTT | 1.0 |
| delTACA | 1.0 |
| dupA | 3.0 |
| dupAA | 1.0 |
| dupAAGT | 1.0 |
| dupG | 1.0 |
| dupT | 2.0 |
| insC | 1.0 |
| insT | 1.0 |
| indel | 1.0 |
