# Supplementary material for: Comprehensive genetic analysis of 961 unrelated Duchenne Muscular Dystrophy patients: Focus on diagnosis, prevention and therapeutic possibilities
Source: PLoS One. 2020 Jun 19;15(6):e0232654. doi: 10.1371/journal.pone.0232654 (PMC7304910; doi:10.1371/journal.pone.0232654)
Supplement: S4 Fig — (PPTX) [file pone.0232654.s004.pptx]

## Slide 1
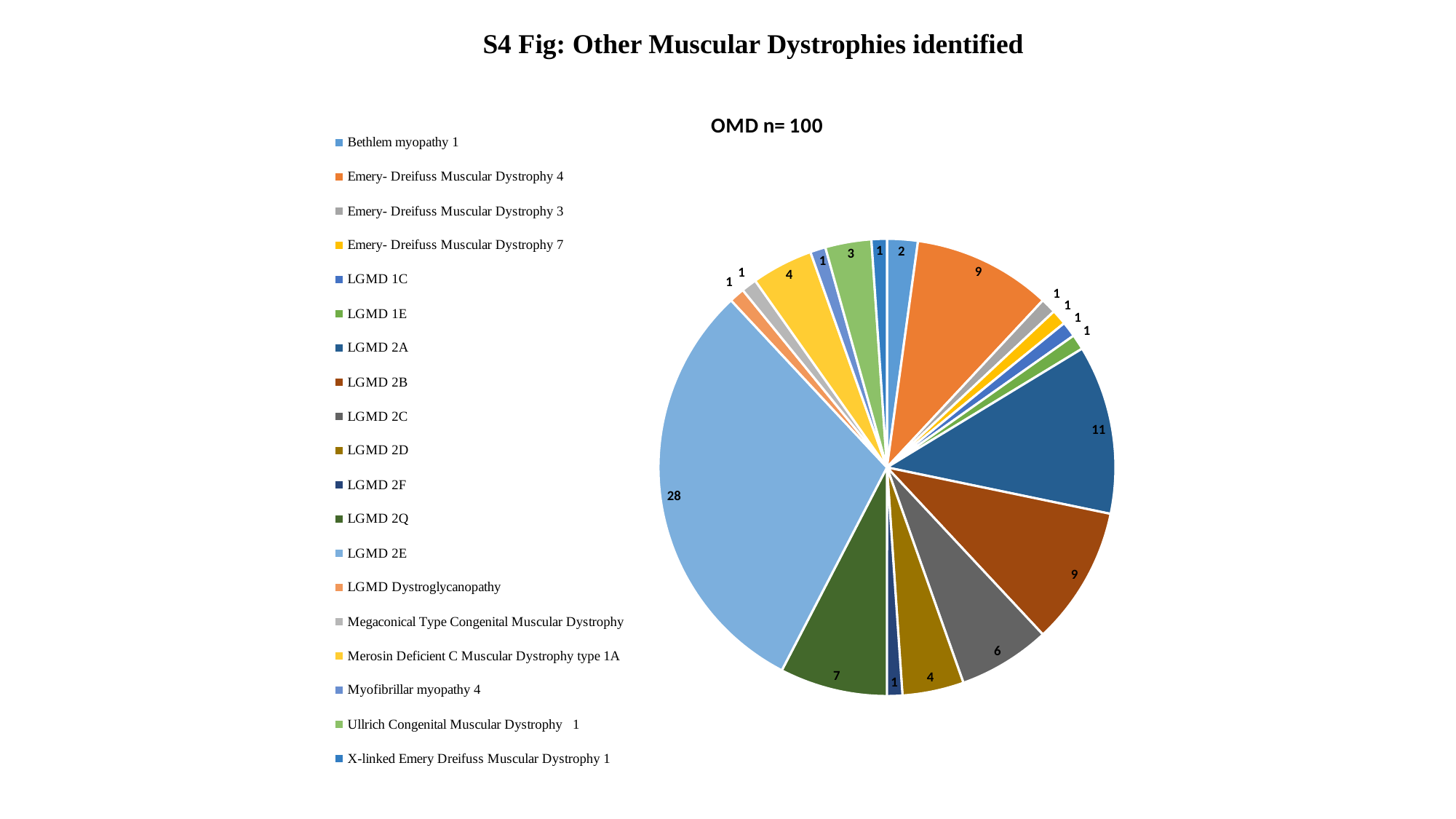

S4 Fig: Other Muscular Dystrophies identified
### Chart: OMD n= 100
| Category | Total No.s |
|---|---|
| Bethlem myopathy 1 | 2.0 |
| Emery- Dreifuss Muscular Dystrophy 4 | 9.0 |
| Emery- Dreifuss Muscular Dystrophy 3 | 1.0 |
| Emery- Dreifuss Muscular Dystrophy 7 | 1.0 |
| LGMD 1C | 1.0 |
| LGMD 1E | 1.0 |
| LGMD 2A | 11.0 |
| LGMD 2B | 9.0 |
| LGMD 2C | 6.0 |
| LGMD 2D | 4.0 |
| LGMD 2F | 1.0 |
| LGMD 2Q | 7.0 |
| LGMD 2E | 28.0 |
| LGMD Dystroglycanopathy | 1.0 |
| Megaconical Type Congenital Muscular Dystrophy | 1.0 |
| Merosin Deficient C Muscular Dystrophy type 1A | 4.0 |
| Myofibrillar myopathy 4 | 1.0 |
| Ullrich Congenital Muscular Dystrophy 1 | 3.0 |
| X-linked Emery Dreifuss Muscular Dystrophy 1 | 1.0 |
