# Supplementary material for: Comprehensive genetic analysis of 961 unrelated Duchenne Muscular Dystrophy patients: Focus on diagnosis, prevention and therapeutic possibilities
Source: PLoS One. 2020 Jun 19;15(6):e0232654. doi: 10.1371/journal.pone.0232654 (PMC7304910; doi:10.1371/journal.pone.0232654)
Supplement: S1 Table — (PPTX) [file pone.0232654.s005.pptx]

## Slide 1
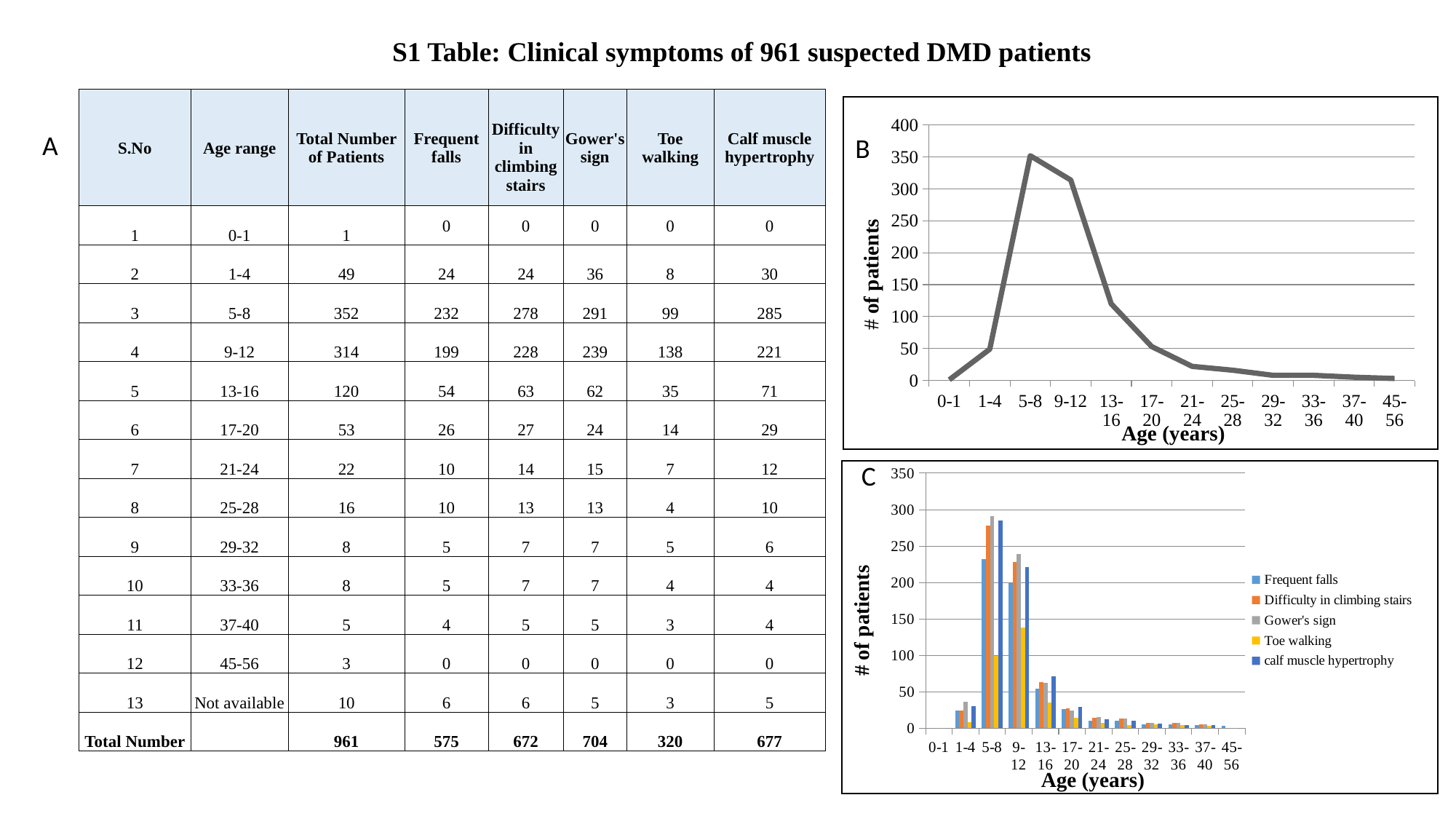

S1 Table: Clinical symptoms of 961 suspected DMD patients
| S.No | Age range | Total Number of Patients | Frequent falls | Difficulty in climbing stairs | Gower's sign | Toe walking | Calf muscle hypertrophy |
| --- | --- | --- | --- | --- | --- | --- | --- |
| 1 | 0-1 | 1 | 0 | 0 | 0 | 0 | 0 |
| 2 | 1-4 | 49 | 24 | 24 | 36 | 8 | 30 |
| 3 | 5-8 | 352 | 232 | 278 | 291 | 99 | 285 |
| 4 | 9-12 | 314 | 199 | 228 | 239 | 138 | 221 |
| 5 | 13-16 | 120 | 54 | 63 | 62 | 35 | 71 |
| 6 | 17-20 | 53 | 26 | 27 | 24 | 14 | 29 |
| 7 | 21-24 | 22 | 10 | 14 | 15 | 7 | 12 |
| 8 | 25-28 | 16 | 10 | 13 | 13 | 4 | 10 |
| 9 | 29-32 | 8 | 5 | 7 | 7 | 5 | 6 |
| 10 | 33-36 | 8 | 5 | 7 | 7 | 4 | 4 |
| 11 | 37-40 | 5 | 4 | 5 | 5 | 3 | 4 |
| 12 | 45-56 | 3 | 0 | 0 | 0 | 0 | 0 |
| 13 | Not available | 10 | 6 | 6 | 5 | 3 | 5 |
| Total Number | | 961 | 575 | 672 | 704 | 320 | 677 |
### Chart
| Category | Total Number of Patients |
|---|---|
| 0-1 | 1.0 |
| 1-4 | 49.0 |
| 5-8 | 352.0 |
| 9-12 | 314.0 |
| 13-16 | 120.0 |
| 17-20 | 53.0 |
| 21-24 | 22.0 |
| 25-28 | 16.0 |
| 29-32 | 8.0 |
| 33-36 | 8.0 |
| 37-40 | 5.0 |
| 45-56 | 3.0 |A
B
# of patients
Age (years)
C
### Chart
| Category | Frequent falls | Difficulty in climbing stairs | Gower's sign | Toe walking | calf muscle hypertrophy |
|---|---|---|---|---|---|
| 0-1 | 0.0 | 0.0 | 0.0 | 0.0 | 0.0 |
| 1-4 | 24.0 | 24.0 | 36.0 | 8.0 | 30.0 |
| 5-8 | 232.0 | 278.0 | 291.0 | 99.0 | 285.0 |
| 9-12 | 199.0 | 228.0 | 239.0 | 138.0 | 221.0 |
| 13-16 | 54.0 | 63.0 | 62.0 | 35.0 | 71.0 |
| 17-20 | 26.0 | 27.0 | 24.0 | 14.0 | 29.0 |
| 21-24 | 10.0 | 14.0 | 15.0 | 7.0 | 12.0 |
| 25-28 | 10.0 | 13.0 | 13.0 | 4.0 | 10.0 |
| 29-32 | 5.0 | 7.0 | 7.0 | 5.0 | 6.0 |
| 33-36 | 5.0 | 7.0 | 7.0 | 4.0 | 4.0 |
| 37-40 | 4.0 | 5.0 | 5.0 | 3.0 | 4.0 |
| 45-56 | 3.0 | 0.0 | 0.0 | 0.0 | 0.0 |
# of patients
Age (years)
