# Supplementary material for: Comprehensive genetic analysis of 961 unrelated Duchenne Muscular Dystrophy patients: Focus on diagnosis, prevention and therapeutic possibilities
Source: PLoS One. 2020 Jun 19;15(6):e0232654. doi: 10.1371/journal.pone.0232654 (PMC7304910; doi:10.1371/journal.pone.0232654)
Supplement: S2 Table — (PPTX) [file pone.0232654.s006.pptx]

## Slide 1
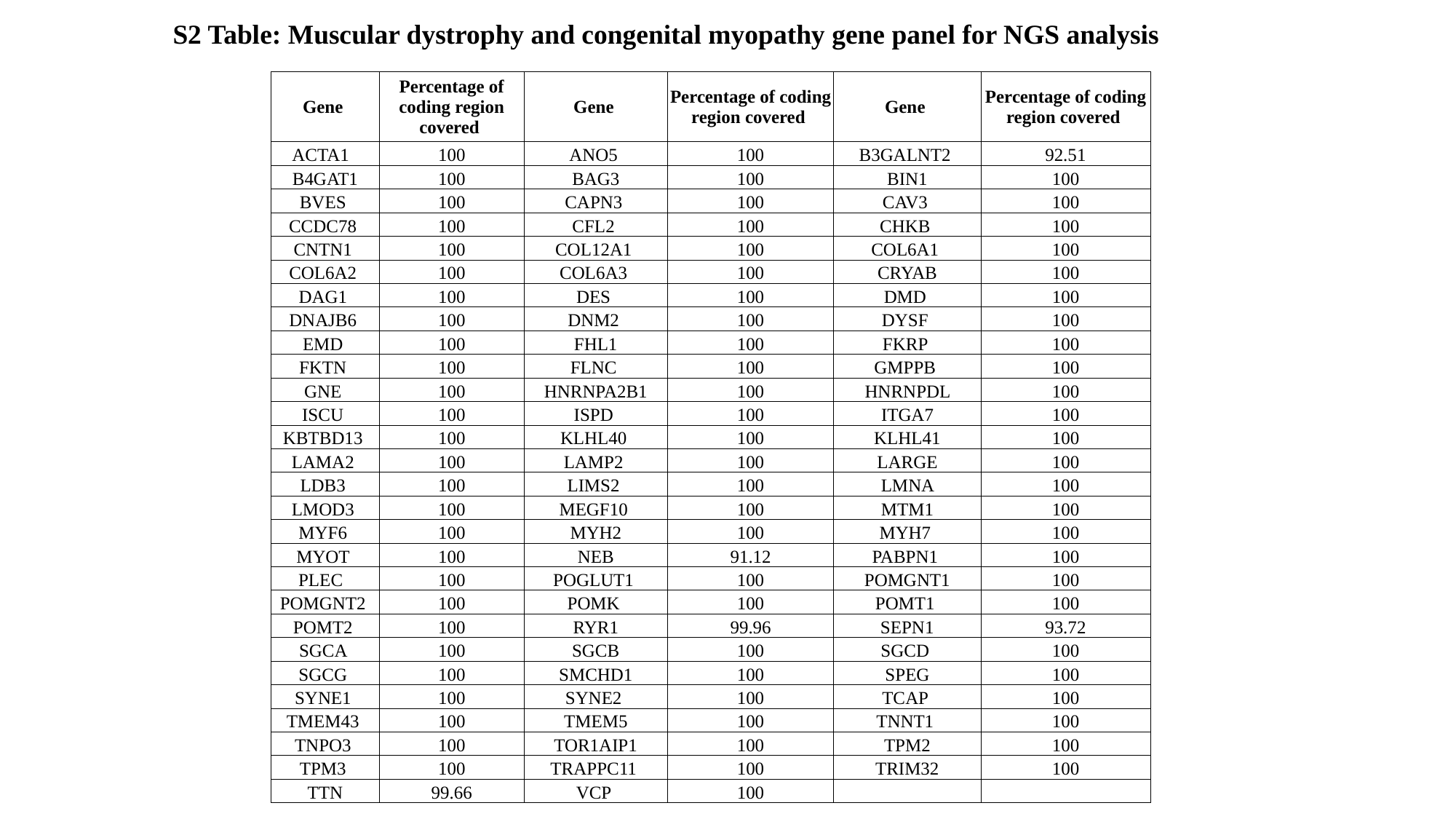

S2 Table: Muscular dystrophy and congenital myopathy gene panel for NGS analysis
| Gene | Percentage of coding region covered | Gene | Percentage of coding region covered | Gene | Percentage of coding region covered |
| --- | --- | --- | --- | --- | --- |
| ACTA1 | 100 | ANO5 | 100 | B3GALNT2 | 92.51 |
| B4GAT1 | 100 | BAG3 | 100 | BIN1 | 100 |
| BVES | 100 | CAPN3 | 100 | CAV3 | 100 |
| CCDC78 | 100 | CFL2 | 100 | CHKB | 100 |
| CNTN1 | 100 | COL12A1 | 100 | COL6A1 | 100 |
| COL6A2 | 100 | COL6A3 | 100 | CRYAB | 100 |
| DAG1 | 100 | DES | 100 | DMD | 100 |
| DNAJB6 | 100 | DNM2 | 100 | DYSF | 100 |
| EMD | 100 | FHL1 | 100 | FKRP | 100 |
| FKTN | 100 | FLNC | 100 | GMPPB | 100 |
| GNE | 100 | HNRNPA2B1 | 100 | HNRNPDL | 100 |
| ISCU | 100 | ISPD | 100 | ITGA7 | 100 |
| KBTBD13 | 100 | KLHL40 | 100 | KLHL41 | 100 |
| LAMA2 | 100 | LAMP2 | 100 | LARGE | 100 |
| LDB3 | 100 | LIMS2 | 100 | LMNA | 100 |
| LMOD3 | 100 | MEGF10 | 100 | MTM1 | 100 |
| MYF6 | 100 | MYH2 | 100 | MYH7 | 100 |
| MYOT | 100 | NEB | 91.12 | PABPN1 | 100 |
| PLEC | 100 | POGLUT1 | 100 | POMGNT1 | 100 |
| POMGNT2 | 100 | POMK | 100 | POMT1 | 100 |
| POMT2 | 100 | RYR1 | 99.96 | SEPN1 | 93.72 |
| SGCA | 100 | SGCB | 100 | SGCD | 100 |
| SGCG | 100 | SMCHD1 | 100 | SPEG | 100 |
| SYNE1 | 100 | SYNE2 | 100 | TCAP | 100 |
| TMEM43 | 100 | TMEM5 | 100 | TNNT1 | 100 |
| TNPO3 | 100 | TOR1AIP1 | 100 | TPM2 | 100 |
| TPM3 | 100 | TRAPPC11 | 100 | TRIM32 | 100 |
| TTN | 99.66 | VCP | 100 | | |
