# Supplementary material for: Comprehensive genetic analysis of 961 unrelated Duchenne Muscular Dystrophy patients: Focus on diagnosis, prevention and therapeutic possibilities
Source: PLoS One. 2020 Jun 19;15(6):e0232654. doi: 10.1371/journal.pone.0232654 (PMC7304910; doi:10.1371/journal.pone.0232654)
Supplement: S3 Table — (PPTX) [file pone.0232654.s007.pptx]

## Slide 1
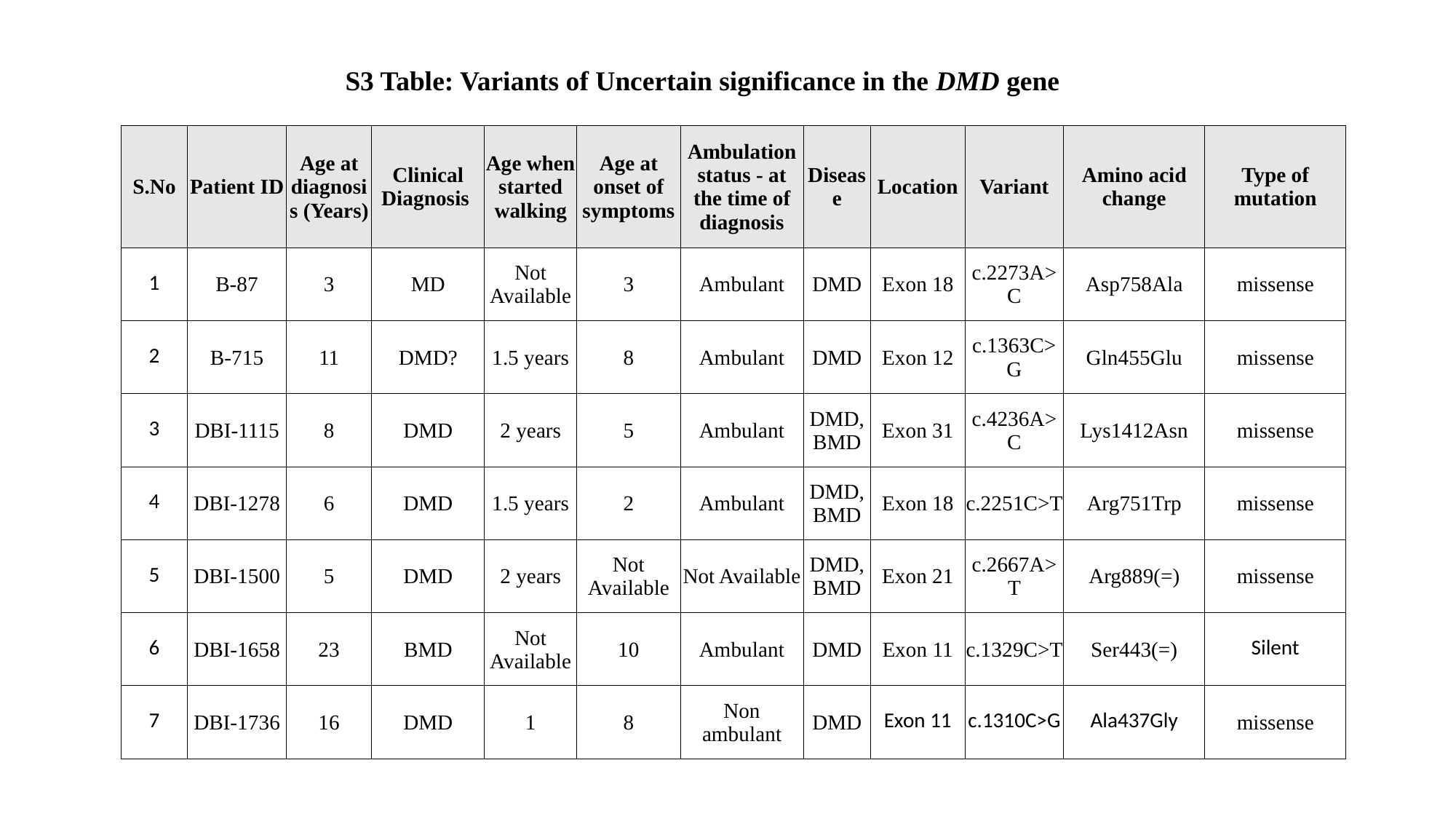

S3 Table: Variants of Uncertain significance in the DMD gene
| S.No | Patient ID | Age at diagnosis (Years) | Clinical Diagnosis | Age when started walking | Age at onset of symptoms | Ambulation status - at the time of diagnosis | Disease | Location | Variant | Amino acid change | Type of mutation |
| --- | --- | --- | --- | --- | --- | --- | --- | --- | --- | --- | --- |
| 1 | B-87 | 3 | MD | Not Available | 3 | Ambulant | DMD | Exon 18 | c.2273A>C | Asp758Ala | missense |
| 2 | B-715 | 11 | DMD? | 1.5 years | 8 | Ambulant | DMD | Exon 12 | c.1363C>G | Gln455Glu | missense |
| 3 | DBI-1115 | 8 | DMD | 2 years | 5 | Ambulant | DMD, BMD | Exon 31 | c.4236A>C | Lys1412Asn | missense |
| 4 | DBI-1278 | 6 | DMD | 1.5 years | 2 | Ambulant | DMD, BMD | Exon 18 | c.2251C>T | Arg751Trp | missense |
| 5 | DBI-1500 | 5 | DMD | 2 years | Not Available | Not Available | DMD, BMD | Exon 21 | c.2667A>T | Arg889(=) | missense |
| 6 | DBI-1658 | 23 | BMD | Not Available | 10 | Ambulant | DMD | Exon 11 | c.1329C>T | Ser443(=) | Silent |
| 7 | DBI-1736 | 16 | DMD | 1 | 8 | Non ambulant | DMD | Exon 11 | c.1310C>G | Ala437Gly | missense |
