# Supplementary material for: Comprehensive genetic analysis of 961 unrelated Duchenne Muscular Dystrophy patients: Focus on diagnosis, prevention and therapeutic possibilities
Source: PLoS One. 2020 Jun 19;15(6):e0232654. doi: 10.1371/journal.pone.0232654 (PMC7304910; doi:10.1371/journal.pone.0232654)
Supplement: S4 Table — (PPTX) [file pone.0232654.s008.pptx]

## Slide 1
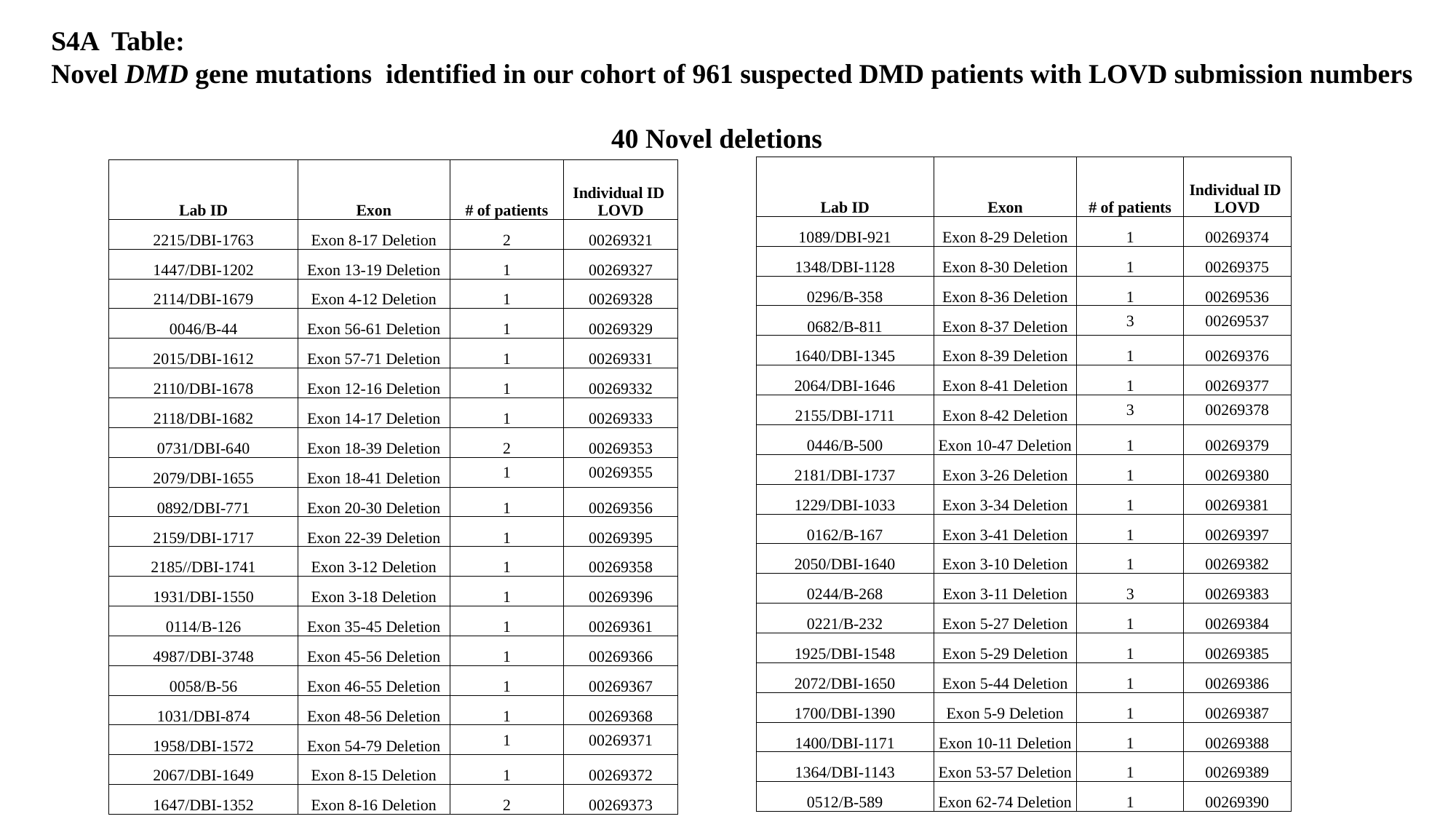

S4A Table:
Novel DMD gene mutations identified in our cohort of 961 suspected DMD patients with LOVD submission numbers
40 Novel deletions
| Lab ID | Exon | # of patients | Individual ID LOVD |
| --- | --- | --- | --- |
| 1089/DBI-921 | Exon 8-29 Deletion | 1 | 00269374 |
| 1348/DBI-1128 | Exon 8-30 Deletion | 1 | 00269375 |
| 0296/B-358 | Exon 8-36 Deletion | 1 | 00269536 |
| 0682/B-811 | Exon 8-37 Deletion | 3 | 00269537 |
| 1640/DBI-1345 | Exon 8-39 Deletion | 1 | 00269376 |
| 2064/DBI-1646 | Exon 8-41 Deletion | 1 | 00269377 |
| 2155/DBI-1711 | Exon 8-42 Deletion | 3 | 00269378 |
| 0446/B-500 | Exon 10-47 Deletion | 1 | 00269379 |
| 2181/DBI-1737 | Exon 3-26 Deletion | 1 | 00269380 |
| 1229/DBI-1033 | Exon 3-34 Deletion | 1 | 00269381 |
| 0162/B-167 | Exon 3-41 Deletion | 1 | 00269397 |
| 2050/DBI-1640 | Exon 3-10 Deletion | 1 | 00269382 |
| 0244/B-268 | Exon 3-11 Deletion | 3 | 00269383 |
| 0221/B-232 | Exon 5-27 Deletion | 1 | 00269384 |
| 1925/DBI-1548 | Exon 5-29 Deletion | 1 | 00269385 |
| 2072/DBI-1650 | Exon 5-44 Deletion | 1 | 00269386 |
| 1700/DBI-1390 | Exon 5-9 Deletion | 1 | 00269387 |
| 1400/DBI-1171 | Exon 10-11 Deletion | 1 | 00269388 |
| 1364/DBI-1143 | Exon 53-57 Deletion | 1 | 00269389 |
| 0512/B-589 | Exon 62-74 Deletion | 1 | 00269390 |
| Lab ID | Exon | # of patients | Individual ID LOVD |
| --- | --- | --- | --- |
| 2215/DBI-1763 | Exon 8-17 Deletion | 2 | 00269321 |
| 1447/DBI-1202 | Exon 13-19 Deletion | 1 | 00269327 |
| 2114/DBI-1679 | Exon 4-12 Deletion | 1 | 00269328 |
| 0046/B-44 | Exon 56-61 Deletion | 1 | 00269329 |
| 2015/DBI-1612 | Exon 57-71 Deletion | 1 | 00269331 |
| 2110/DBI-1678 | Exon 12-16 Deletion | 1 | 00269332 |
| 2118/DBI-1682 | Exon 14-17 Deletion | 1 | 00269333 |
| 0731/DBI-640 | Exon 18-39 Deletion | 2 | 00269353 |
| 2079/DBI-1655 | Exon 18-41 Deletion | 1 | 00269355 |
| 0892/DBI-771 | Exon 20-30 Deletion | 1 | 00269356 |
| 2159/DBI-1717 | Exon 22-39 Deletion | 1 | 00269395 |
| 2185//DBI-1741 | Exon 3-12 Deletion | 1 | 00269358 |
| 1931/DBI-1550 | Exon 3-18 Deletion | 1 | 00269396 |
| 0114/B-126 | Exon 35-45 Deletion | 1 | 00269361 |
| 4987/DBI-3748 | Exon 45-56 Deletion | 1 | 00269366 |
| 0058/B-56 | Exon 46-55 Deletion | 1 | 00269367 |
| 1031/DBI-874 | Exon 48-56 Deletion | 1 | 00269368 |
| 1958/DBI-1572 | Exon 54-79 Deletion | 1 | 00269371 |
| 2067/DBI-1649 | Exon 8-15 Deletion | 1 | 00269372 |
| 1647/DBI-1352 | Exon 8-16 Deletion | 2 | 00269373 |

## Slide 2
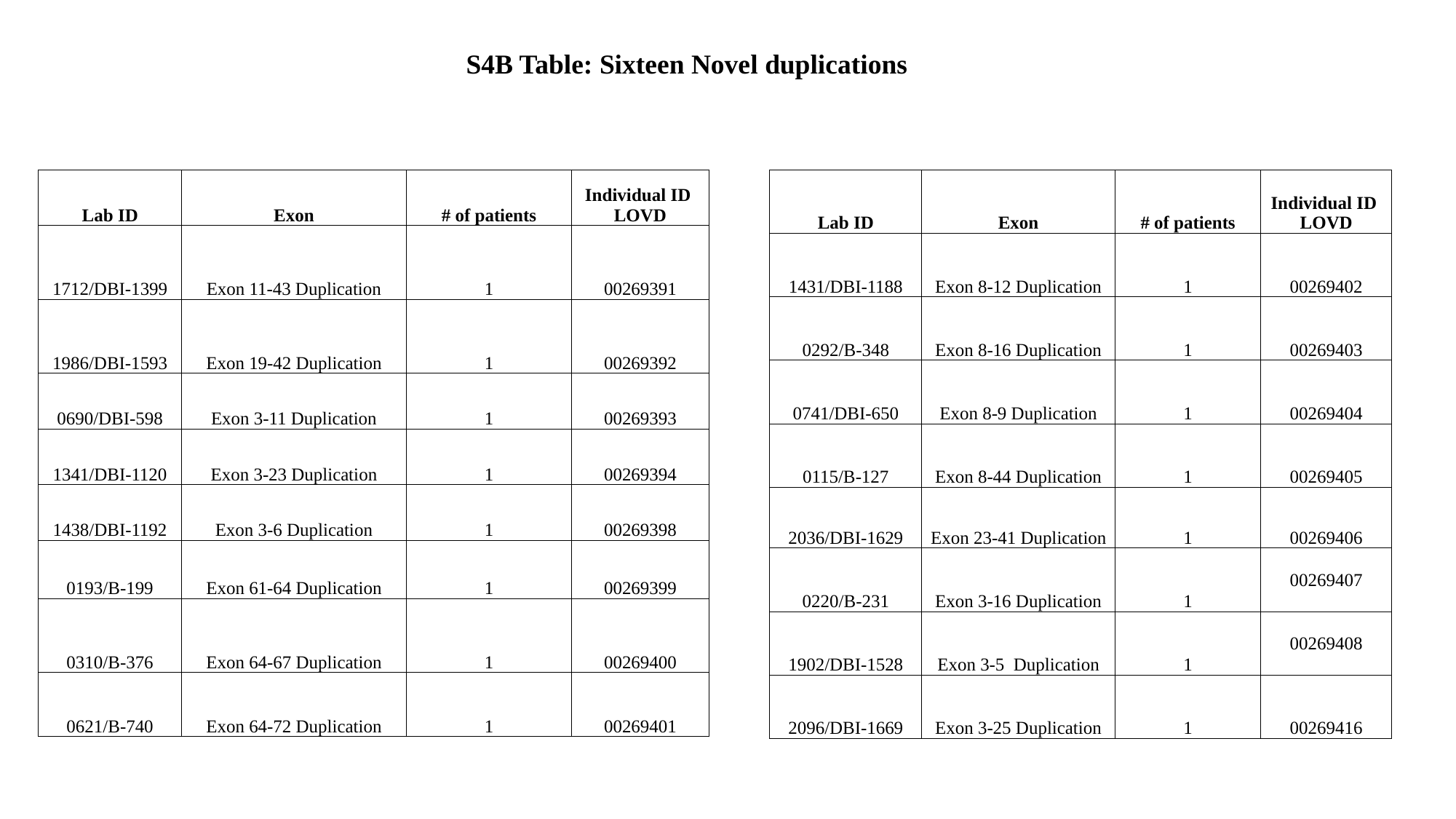

S4B Table: Sixteen Novel duplications
| Lab ID | Exon | # of patients | Individual ID LOVD |
| --- | --- | --- | --- |
| 1712/DBI-1399 | Exon 11-43 Duplication | 1 | 00269391 |
| 1986/DBI-1593 | Exon 19-42 Duplication | 1 | 00269392 |
| 0690/DBI-598 | Exon 3-11 Duplication | 1 | 00269393 |
| 1341/DBI-1120 | Exon 3-23 Duplication | 1 | 00269394 |
| 1438/DBI-1192 | Exon 3-6 Duplication | 1 | 00269398 |
| 0193/B-199 | Exon 61-64 Duplication | 1 | 00269399 |
| 0310/B-376 | Exon 64-67 Duplication | 1 | 00269400 |
| 0621/B-740 | Exon 64-72 Duplication | 1 | 00269401 |
| Lab ID | Exon | # of patients | Individual ID LOVD |
| --- | --- | --- | --- |
| 1431/DBI-1188 | Exon 8-12 Duplication | 1 | 00269402 |
| 0292/B-348 | Exon 8-16 Duplication | 1 | 00269403 |
| 0741/DBI-650 | Exon 8-9 Duplication | 1 | 00269404 |
| 0115/B-127 | Exon 8-44 Duplication | 1 | 00269405 |
| 2036/DBI-1629 | Exon 23-41 Duplication | 1 | 00269406 |
| 0220/B-231 | Exon 3-16 Duplication | 1 | 00269407 |
| 1902/DBI-1528 | Exon 3-5 Duplication | 1 | 00269408 |
| 2096/DBI-1669 | Exon 3-25 Duplication | 1 | 00269416 |

## Slide 3
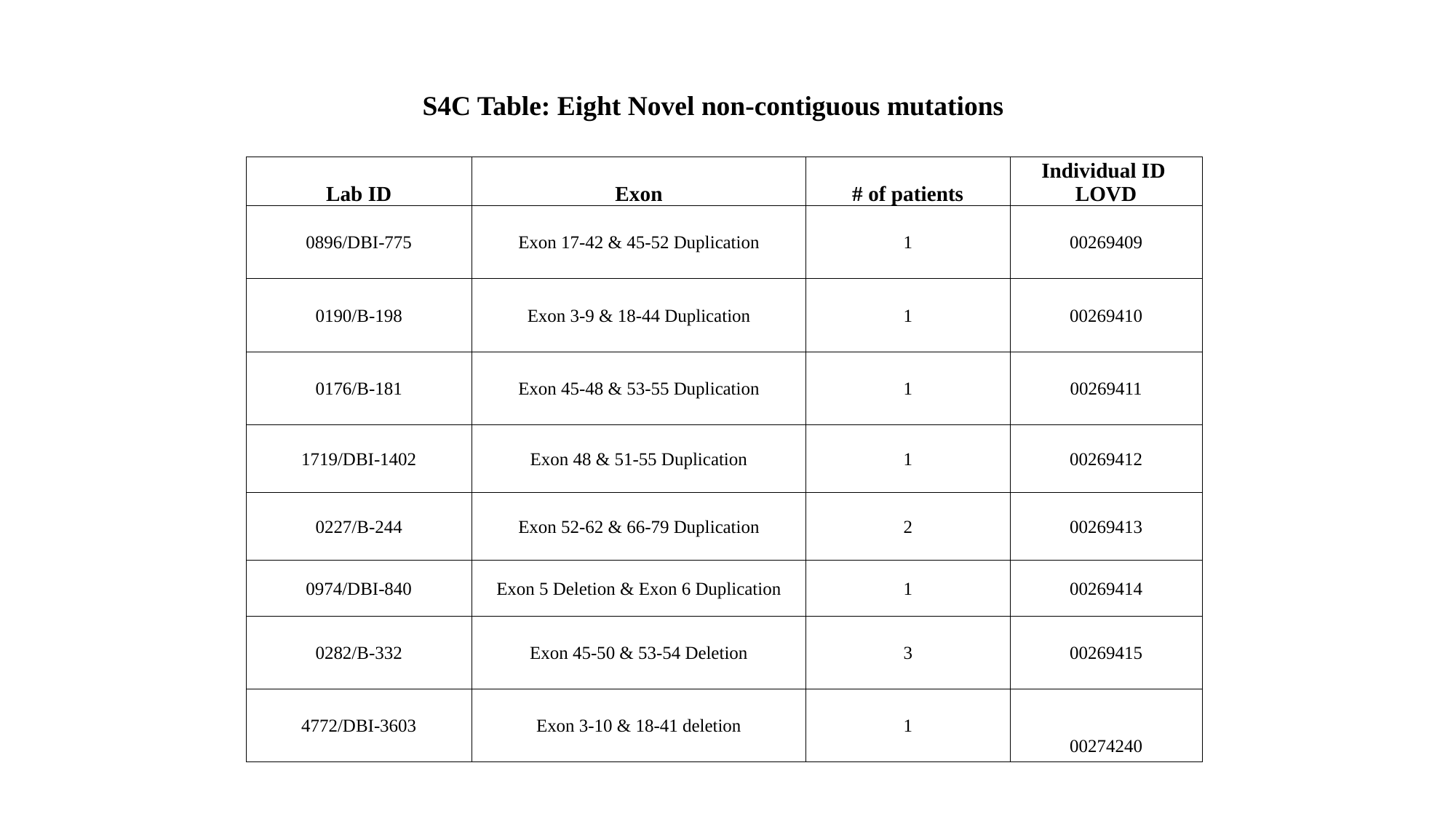

S4C Table: Eight Novel non-contiguous mutations
| Lab ID | Exon | # of patients | Individual ID LOVD |
| --- | --- | --- | --- |
| 0896/DBI-775 | Exon 17-42 & 45-52 Duplication | 1 | 00269409 |
| 0190/B-198 | Exon 3-9 & 18-44 Duplication | 1 | 00269410 |
| 0176/B-181 | Exon 45-48 & 53-55 Duplication | 1 | 00269411 |
| 1719/DBI-1402 | Exon 48 & 51-55 Duplication | 1 | 00269412 |
| 0227/B-244 | Exon 52-62 & 66-79 Duplication | 2 | 00269413 |
| 0974/DBI-840 | Exon 5 Deletion & Exon 6 Duplication | 1 | 00269414 |
| 0282/B-332 | Exon 45-50 & 53-54 Deletion | 3 | 00269415 |
| 4772/DBI-3603 | Exon 3-10 & 18-41 deletion | 1 | 00274240 |
