# Supplementary material for: Comprehensive genetic analysis of 961 unrelated Duchenne Muscular Dystrophy patients: Focus on diagnosis, prevention and therapeutic possibilities
Source: PLoS One. 2020 Jun 19;15(6):e0232654. doi: 10.1371/journal.pone.0232654 (PMC7304910; doi:10.1371/journal.pone.0232654)
Supplement: S5 Table — (PPTX) [file pone.0232654.s009.pptx]

## Slide 1
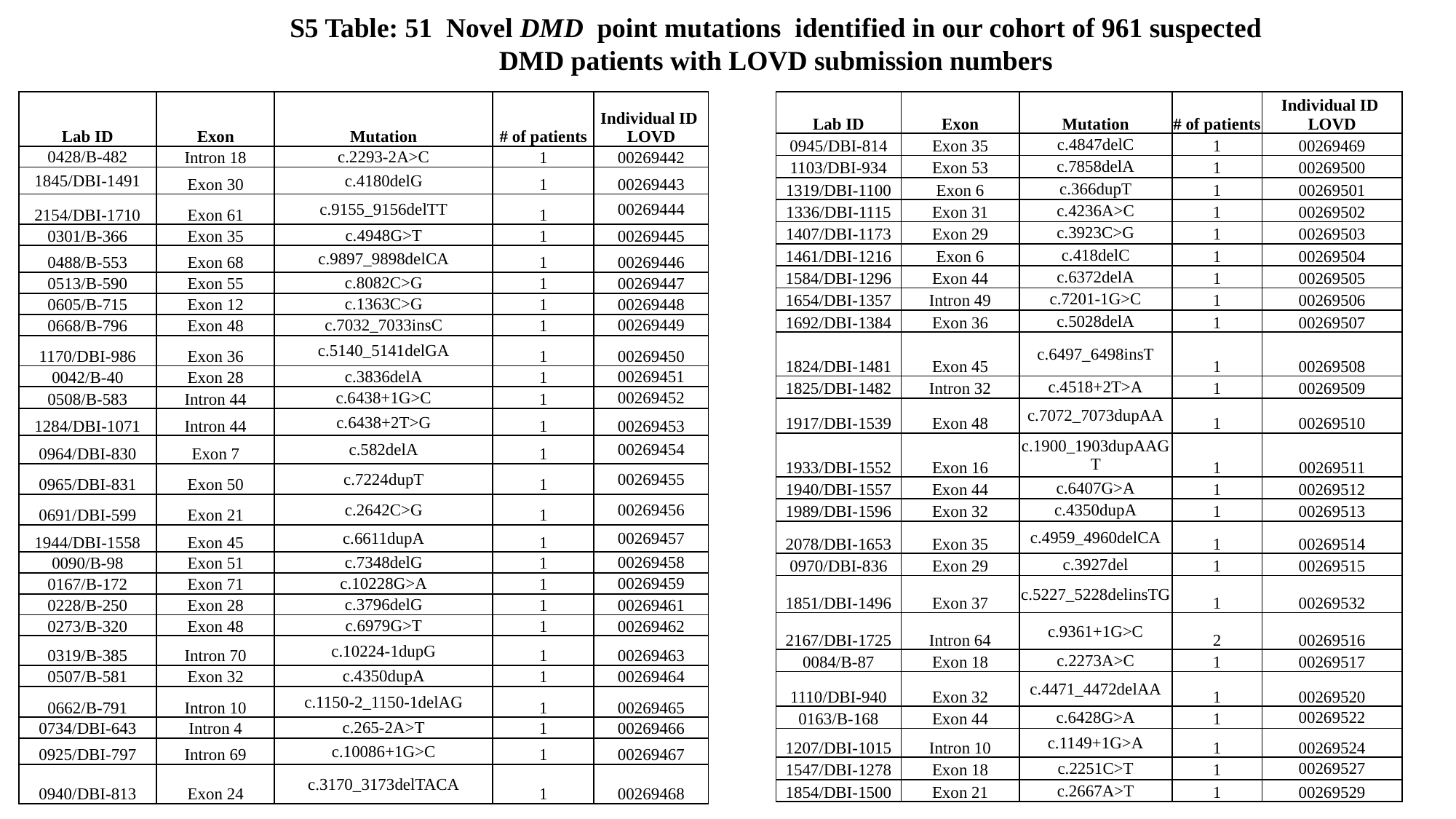

S5 Table: 51 Novel DMD point mutations identified in our cohort of 961 suspected DMD patients with LOVD submission numbers
| Lab ID | Exon | Mutation | # of patients | Individual ID LOVD |
| --- | --- | --- | --- | --- |
| 0428/B-482 | Intron 18 | c.2293-2A>C | 1 | 00269442 |
| 1845/DBI-1491 | Exon 30 | c.4180delG | 1 | 00269443 |
| 2154/DBI-1710 | Exon 61 | c.9155\_9156delTT | 1 | 00269444 |
| 0301/B-366 | Exon 35 | c.4948G>T | 1 | 00269445 |
| 0488/B-553 | Exon 68 | c.9897\_9898delCA | 1 | 00269446 |
| 0513/B-590 | Exon 55 | c.8082C>G | 1 | 00269447 |
| 0605/B-715 | Exon 12 | c.1363C>G | 1 | 00269448 |
| 0668/B-796 | Exon 48 | c.7032\_7033insC | 1 | 00269449 |
| 1170/DBI-986 | Exon 36 | c.5140\_5141delGA | 1 | 00269450 |
| 0042/B-40 | Exon 28 | c.3836delA | 1 | 00269451 |
| 0508/B-583 | Intron 44 | c.6438+1G>C | 1 | 00269452 |
| 1284/DBI-1071 | Intron 44 | c.6438+2T>G | 1 | 00269453 |
| 0964/DBI-830 | Exon 7 | c.582delA | 1 | 00269454 |
| 0965/DBI-831 | Exon 50 | c.7224dupT | 1 | 00269455 |
| 0691/DBI-599 | Exon 21 | c.2642C>G | 1 | 00269456 |
| 1944/DBI-1558 | Exon 45 | c.6611dupA | 1 | 00269457 |
| 0090/B-98 | Exon 51 | c.7348delG | 1 | 00269458 |
| 0167/B-172 | Exon 71 | c.10228G>A | 1 | 00269459 |
| 0228/B-250 | Exon 28 | c.3796delG | 1 | 00269461 |
| 0273/B-320 | Exon 48 | c.6979G>T | 1 | 00269462 |
| 0319/B-385 | Intron 70 | c.10224-1dupG | 1 | 00269463 |
| 0507/B-581 | Exon 32 | c.4350dupA | 1 | 00269464 |
| 0662/B-791 | Intron 10 | c.1150-2\_1150-1delAG | 1 | 00269465 |
| 0734/DBI-643 | Intron 4 | c.265-2A>T | 1 | 00269466 |
| 0925/DBI-797 | Intron 69 | c.10086+1G>C | 1 | 00269467 |
| 0940/DBI-813 | Exon 24 | c.3170\_3173delTACA | 1 | 00269468 |
| Lab ID | Exon | Mutation | # of patients | Individual ID LOVD |
| --- | --- | --- | --- | --- |
| 0945/DBI-814 | Exon 35 | c.4847delC | 1 | 00269469 |
| 1103/DBI-934 | Exon 53 | c.7858delA | 1 | 00269500 |
| 1319/DBI-1100 | Exon 6 | c.366dupT | 1 | 00269501 |
| 1336/DBI-1115 | Exon 31 | c.4236A>C | 1 | 00269502 |
| 1407/DBI-1173 | Exon 29 | c.3923C>G | 1 | 00269503 |
| 1461/DBI-1216 | Exon 6 | c.418delC | 1 | 00269504 |
| 1584/DBI-1296 | Exon 44 | c.6372delA | 1 | 00269505 |
| 1654/DBI-1357 | Intron 49 | c.7201-1G>C | 1 | 00269506 |
| 1692/DBI-1384 | Exon 36 | c.5028delA | 1 | 00269507 |
| 1824/DBI-1481 | Exon 45 | c.6497\_6498insT | 1 | 00269508 |
| 1825/DBI-1482 | Intron 32 | c.4518+2T>A | 1 | 00269509 |
| 1917/DBI-1539 | Exon 48 | c.7072\_7073dupAA | 1 | 00269510 |
| 1933/DBI-1552 | Exon 16 | c.1900\_1903dupAAGT | 1 | 00269511 |
| 1940/DBI-1557 | Exon 44 | c.6407G>A | 1 | 00269512 |
| 1989/DBI-1596 | Exon 32 | c.4350dupA | 1 | 00269513 |
| 2078/DBI-1653 | Exon 35 | c.4959\_4960delCA | 1 | 00269514 |
| 0970/DBI-836 | Exon 29 | c.3927del | 1 | 00269515 |
| 1851/DBI-1496 | Exon 37 | c.5227\_5228delinsTG | 1 | 00269532 |
| 2167/DBI-1725 | Intron 64 | c.9361+1G>C | 2 | 00269516 |
| 0084/B-87 | Exon 18 | c.2273A>C | 1 | 00269517 |
| 1110/DBI-940 | Exon 32 | c.4471\_4472delAA | 1 | 00269520 |
| 0163/B-168 | Exon 44 | c.6428G>A | 1 | 00269522 |
| 1207/DBI-1015 | Intron 10 | c.1149+1G>A | 1 | 00269524 |
| 1547/DBI-1278 | Exon 18 | c.2251C>T | 1 | 00269527 |
| 1854/DBI-1500 | Exon 21 | c.2667A>T | 1 | 00269529 |
